# Microplastic effects on soil nitrogen storage, nitrogen emissions, and ammonia volatilization in relation to soil health and crop productivity: mechanism and future consideration

**DOI:** 10.3389/fpls.2025.1621542

**Published:** 2025-09-29

**Authors:** Umair Sarfraz, Yinsen Qian, Qiaoqiao Yu, Yifan Cao, Xiaoyi Jiang, Nida Mahreen, Rongrong Tao, Quan Ma, Min Zhu, Jinfeng Ding, Chunyan Li, Wenshan Guo, Xinkai Zhu

**Affiliations:** ^1^ Jiangsu Key Laboratory of Crop Genetics and Physiology, Agricultural College of Yangzhou University, Yangzhou, Jiangsu, China; ^2^ Horticulture Research Institute, Ayyub Agriculture Research Institute, Faisalabad, Punjab, Pakistan; ^3^ Co-Innovation Center for Modern Production Technology of Grain Crops, Yangzhou University, Yangzhou, Jiangsu, China; ^4^ Joint International Research Laboratory of Agriculture and Agri-Product Safety, the Ministry of Education of China, Yangzhou University, Yangzhou, Jiangsu, China

**Keywords:** microplastic pollution, nitrogen cycle, ammonia volatilization, soil health, biochar remediation

## Abstract

Microplastic contamination in agricultural soils is emerging as a significant environmental challenge due to its detrimental effects on soil health, nitrogen cycling, and crop productivity. This review paper synthesizes current knowledge on the impacts of various microplastics, specifically polyethylene (PE), polyvinyl chloride (PVC), and polypropylene (PP), on agricultural systems, with a particular focus on their interactions with nitrogen dynamics and ammonia volatilization processes. Microplastics enter agricultural soils through multiple sources, including plastic mulching, irrigation, and application of biosolids, leading to alterations in soil physical and chemical properties, nutrient availability, and microbial activity. These changes negatively influence critical soil processes such as nitrogen mineralization, nitrification, and denitrification, thereby reducing nitrogen use efficiency (NUE) and increasing ammonia volatilization. Consequently, these disturbances manifest in reduced crop growth and productivity, particularly affecting crops such as wheat. This review also explores biochar as a promising remediation strategy, highlighting its potential to mitigate microplastic-induced disruptions in soil ecosystems by improving soil structure, enhancing nitrogen retention, and reducing ammonia emissions. However, the paper identifies significant knowledge gaps, including the need for standardized methodologies and long-term field studies to understand the cumulative impacts of microplastics comprehensively. To address microplastic pollution effectively, integrated approaches combining scientific research, sustainable agricultural practices, and robust policy frameworks are recommended. This will ensure agricultural sustainability, soil fertility, and food security amidst growing environmental concerns.

## 1 Introduction

The extensive use of plastic in agriculture and the degradation of larger plastic objects are the sources of microplastics, which are particles of plastic less than 5 mm in size. Their bad impact on the ecosystem has the potential to diminish soil quality and reduce agricultural yields (Dindar, 2024). The accumulation of microplastics in agricultural soils is a rising environmental issue, adding to the already substantial problem of microplastic contamination. According to Huang et al. (2023), microplastics are present in many farmlands, and they may damage the soil, disturb plant development, and exacerbate food insecurity. Soil microplastic concentrations in agricultural areas may vary substantially. For instance, according to Bai et al. (2024), there were 1,810 to 86,331 microplastic particles per kilogram of soil in the Hetao Irrigation District of China. This indicates that agricultural practices have a substantial role in the contamination of soil with microplastics (Corradini et al., 2020). Fertilizers, plastic mulch films, sewage sludge, irrigation water, and atmospheric deposition are common sources of microplastics in agricultural soils (Qi et al., 2019a; Surendran et al., 2023) (**Table 1**). The interactions between plants and soil may be drastically altered by even a minute concentration, like 1% of microplastics in soil (Tariq et al., 2024). Soil health and crop development are being affected by the interactions between microplastics and various nitrogen sources, such as organic nitrogen (FYM), polymer-coated urea, biochar-coated urea, and common urea.

**Table 1:** Summary of key studies, research methodologies and main findings.

No.	Scientist (s)	Methodology/research focus	Title of scientist’s paper
1	de Souza MaChado et al. (2019)	Investigated how PE, PVC, and PP microplastics affect soil structure, microbial communities, and enzyme activities involved in nitrogen cycling.	Impacts of microplastics on soil biophysical processes
2	Riveros et al. (2022)	Studied the effects of PP microplastics on ammonium-N levels and nitrification rates, reducing nitrogen availability.	Polypropylene microplastics reduce ammonium-N and nitrification in soils
3	Lu et al. (2021)	Reported enhanced ammonia volatilization due to microplastics, contributing to nitrogen loss in soils.	Microplastics increase ammonia volatilization and nitrogen loss
4	Qi et al. (2019a)	Explored microplastics’ impact on soil redox conditions and nitrogen transformations (nitrification, denitrification).	Microplastic-induced disruption in nitrogen transformations and microbial redox balance
5	Wang et al. (2025)	Analyzed ammonia volatilization and the environmental risks posed by nitrogen loss from soil due to microplastics.	Ammonia volatilization and nitrogen loss under microplastic contamination
6	Sun et al. (2020)	Measured CH_4_ and NH_3_ emissions and compost maturity in relation to PE, PVC, and PHA microplastic presence.	Effects of microplastics on greenhouse gas emissions during composting
7	Guo et al. (2022)	Examined nitrogen uptake disruption in wheat due to functional gene repression and altered microbial diversity.	Microplastic contamination affects nitrogen uptake and functional microbial genes in wheat
8	Jin et al. (2024)	Studied joint toxicity of microplastics and copper ions on plant development and soil microbial health.	Toxic synergy of microplastics and heavy metals on soil health and plants
9	Zhou et al. (2021)	Explored polyhydroxyalkanoates (PHAs) effects on microbial biomass and nutrient turnover in soil hotspots.	Soil microplastic hotspots induced by biodegradable PHAs boost microbial turnover
10	Chen et al. (2023)	Reported GHG emissions and changes in microbial activity with increased PE concentrations in soils.	PE concentration drives GHG emissions and microbial shifts in contaminated soil
11	Zhuang et al. (2025)	Suggested biochar as a remediation strategy to reduce ammonia volatilization and improve nitrogen retention.	Biochar reduces ammonia volatilization in microplastic-contaminated soil
12	Mandal et al. (2015)	Found biochar reduces ammonia volatilization up to 70% by nitrification and NH_3_ adsorption mechanisms.	Mitigating nitrogen loss using biochar: A sustainable soil strategy
13	Zhang et al. (2022)	Studied the effects of microplastics on microbial gene expression, soil enzyme activity, and nitrogen cycling in agricultural soils.	Microplastics impact on soil microbial enzymes and gene suppression
14	Yang et al. (2025)	Investigated interactions of microplastics with cadmium and their combined effect on plant uptake and toxicity.	Combined effects of microplastics and cadmium on plant toxicity
15	Jia et al. (2022)	Measured microplastic accumulation in intensively cultivated agricultural soils.	Assessment of microplastic load in agricultural farmlands

Among the various types of microplastics (MPs) detected in agroecosystems, polyethylene (PE), polyvinyl chloride (PVC), and polypropylene (PP) are among the most frequently found due to their widespread use in agricultural practices such as plastic mulching, irrigation systems, packaging, and greenhouse materials (Piehl et al., 2018; Wang et al., 2021). Their persistence, physicochemical properties, and potential to fragment into smaller particles make them particularly relevant to soil nitrogen processes. For instance, PE is hydrophobic and chemically inert, influencing soil microbial activity and water retention; PVC can release plasticizers and additives that may interfere with microbial nitrogen cycling; while PP, due to its semi-crystalline structure, is more resistant to degradation, potentially causing long-term physical disruption to soil structure (de Souza MaChado et al., 2019; Meng et al., 2020). Compared to polystyrene (PS) or polyethylene terephthalate (PET), which are more prevalent in urban environments, PE, PVC, and PP are more relevant in agricultural contexts, justifying their focused analysis in this review.

The nitrogen cycle, which includes important processes like nitrogen mineralization, nitrification, and denitrification, helps maintain soil fertility and plant growth. Studies show that microplastics can interfere with these processes. For example, polypropylene (PP) microplastics can lower ammonium-N levels and slow down nitrification, reducing the amount of nitrogen available to plants (Riveros et al., 2022). Additionally, microplastics can affect ammonia volatilization, which is when ammonia gas escapes from the soil into the air. This can lead to nitrogen loss, making the soil less fertile and reducing crop yields (Lu et al., 2021). Many factors, such as soil pH, temperature, moisture, and the type of fertilizer used, affect ammonia volatilization (Shan et al., 2015). To reduce nitrogen losses, some management methods, like using biofertilizers, have been found to cut ammonia losses by up to 68% compared to regular fertilizers (Xue et al., 2021). Other methods, such as cover crops and slow-release fertilizers, also help in controlling nitrogen loss (Li et al., 2020a). Biochar-based solutions are also being tested to reduce the harmful effects of microplastics on soil-microbe-plant relationships and to maintain the balance of important nutrients such as carbon (C), nitrogen (N), and phosphorus (P) (Zhang et al., 2022). These studies can help develop better farming methods to deal with microplastic pollution and improve nitrogen use in agriculture, ensuring soil fertility and crop productivity (Zurier and Goddard, 2020). A vital part of soil fertility, the nitrogen cycle, is known to be disrupted by microplastics. They disrupt the microbial populations that transform nitrogen into forms plants can use, known as nitrification and denitrification. Soil nitrogen loss and decreased crop yield are common outcomes of this disturbance, which is accompanied by an increase in ammonia volatilization (Wang et al., 2025; Saqib et al., 2025). The issue of ammonia volatilization poses a serious threat to both agricultural yield and the environment. Air pollution from fine particulate matter, which may harm people’s respiratory health, can result from ammonia loss, which adds to atmospheric nitrogen deposition. Volatilized ammonia, when applied to ecosystems, may worsen soil acidification and eutrophication in bodies of water, leading to ecological imbalances that are harmful to biodiversity and terrestrial life (Lakshmikanthan and Punithavathi, 2024). Secondary air pollutants, such as ammonium nitrate and ammonium sulfate aerosols, are formed in part by ammonia emissions; these aerosols have major effects on public health and climate change (Saqib et al., 2025).

When thinking about sustainable agriculture, the impact of ammonia volatilization on the nitrogen cycle becomes even more apparent. Soil fertility and crop yields are both negatively affected by excessive ammonia losses because less nitrogen is available for plant uptake. Nitrogen deficit stops the growth, biomass output, and grain yields of crops that depend on ammonium and nitrate for growth (Rahut et al., 2025). There is a monetary aspect to this inefficiency as well; farmers have to spend more money on fertilizers to make up for the nitrogen they lose, which drives up production costs and lowers their profit margins.

Environmental and agricultural sustainability depends on the nitrogen cycle and ammonia volatilization. Although ammonia volatilization occurs naturally, it must be controlled to maximize nitrogen use, minimize pollution, and support sustainable agricultural methods. Enhancing soil fertility and crop production, safeguarding ecosystems, and contributing to global food security may all be achieved by addressing nitrogen losses via new approaches and sustainable agriculture management.

Additionally, it has been shown that microplastics might interact with other environmental contaminants, such as cadmium, which could change how plants absorb and react to these pollutants (Yang et al., 2025).

Soil health, nutrient cycling, and crop yield are all negatively impacted by microplastic contamination in agricultural systems, making it a major environmental problem. Plastic mulching, wastewater and biosolids applications, and air deposition are the main entry points for microplastics into agricultural soils (Rahut et al., 2025; Yang et al., 2025). The degradation of plastic mulch, which is used to improve soil water retention and inhibit weed growth, often results in the release of microplastic fragments into the soil. Microplastics are introduced into biosolid applications and wastewater, which are frequently used as fertilizers, since plastics are not completely removed after wastewater treatment (Zhuang et al., 2025).

When microplastics are released into soil, they change their chemical and physical characteristics. Root development and water retention are both hindered as a result of their effect on soil porosity. In addition to their detrimental effects on soil microbes, microplastics may transport harmful substances such as heavy metals and persistent organic pollutants. For example, research has shown that soil deterioration is worsened when copper ions and polystyrene microplastics work together to severely restrict plant development and microbiological activity (Jin et al., 2024). Sun et al. (2025) also discovered that biodegradable microplastics like polylactic acid (PLA) release harmful byproducts during their breakdown, which worsen soil health.

Microplastics and other contaminants have far-reaching consequences that threaten the long-term viability of agriculture. Yuan et al. (2024) and Sun et al. (2025) observed that microplastics may hinder root extension and decrease seed germination rates in pakchoi and rice seedlings, respectively. Also, since microplastics stay in the soil for a long time, their impacts may build up, which is bad news for soil management and our ability to eat.

Microplastics (MPs) have emerged as a ubiquitous environmental pollutant, infiltrating terrestrial ecosystems and posing profound risks to soil health, nutrient cycling, and crop productivity (de Souza MaChado et al., 2018; Rillig, 2012). While the impacts of MPs on aquatic systems have been extensively studied, terrestrial environments, which serve as the ultimate sink for MPs, remain comparatively underexplored. Among the critical soil functions potentially affected, nitrogen (N) cycling is particularly vulnerable due to MPs altering microbial communities, enzyme activities, and nutrient dynamics (Hasan and Tarannum, 2025).

Recent reviews have examined the environmental fate of MPs (Rillig, 2012; Hasan and Tarannum, 2025), yet few have critically synthesized their mechanistic impacts on soil N storage, gaseous N emissions, and ammonia volatilization in the context of soil health and crop production. Moreover, the role of biochar as a potential mitigation strategy against MP contamination has been superficially addressed in earlier works, often without exploring the underlying physicochemical interactions (Zeng et al., 2024).

This review uniquely integrates transcriptomic, biochemical, and physiological insights to unpack how MPs influence soil N dynamics and crop performance. In addition, it critically evaluates biochar’s potential for remediating MP-induced disruptions, providing mechanistic explanations of sorption, stabilization, and ecological trade-offs. This comprehensive synthesis highlights current knowledge gaps, proposes a conceptual framework for MP–N interactions, and outlines future research directions for sustainable soil management under increasing plastic pollution.

Several solutions have been suggested to deal with this increasing problem. According to Rahut et al. (2025), one way to reduce microplastic inputs into agricultural systems is via improved waste management techniques, such as reducing disposable plastics and implementing better recycling methods. Further, there is hope for reducing the effects of microplastics via the creation of sustainable alternatives such as biopolymers and biochar additives. For example, biochar can improve soil structure and stimulate microbial activity, which in turn can help treat soils contaminated with microplastics (Zhuang et al., 2025). The intricate relationships among microplastics, soil ecosystems, and agricultural yields need further study.

### 1.1 Scope and objectives of the review

#### 1.1.1 Scope

In this review, we will look at how microplastic contamination in agricultural soils affects nitrogen cycling and ammonia volatilization. The effects of microplastics like polyethylene (PE), polyvinyl chloride (PVC), and polypropylene (PP) on soil health, agricultural production, and nitrogen dynamics are the main points of this study. This review delves into how microplastics impact vital soil fertility and plant development processes, including nitrification, denitrification, and ammonia volatilization. It also considers how microplastic pollution interacts with various nitrogen fertilizers, such as common urea, urea coated with polymers, urea coated with biochar, and organic nitrogen sources like FYM.

Also included in the assessment are possible ways to lessen the impact of microplastics on farmland soils. We focus on biochar-based remediation solutions because of their ability to improve interactions between soil microbes and plants, keep soil ecosystems balanced, and encourage nutrient cycling. This study will provide policy suggestions for addressing microplastic contamination in farming systems and insights into sustainable agricultural practices by synthesizing existing research.

#### 1.1.2 Objectives

The primary objectives of this review are:

1. To assess the impact of different microplastic types on nitrogen use efficiency and ammonia volatilization in agricultural soils, with a focus on their implications for soil health and crop productivity.
2. To evaluate the effects of microplastic contamination on soil nitrogen transformations under various nitrogen fertilizer sources, including common urea, polymer-coated urea, biochar-coated urea, and organic nitrogen (farm yard manure, FYM).
3. To explore the potential of biochar-based remediation strategies in mitigating the adverse effects of microplastics on soil-microbe-plant interactions and maintaining the nutrient balance, particularly of carbon (C), nitrogen (N), and phosphorus (P) in agricultural systems.

#### 1.1.3 Methodology

Literature used and relevant studies (**Table 1**).

## 2 Microplastic pollution in agriculture

**Figure 1:**
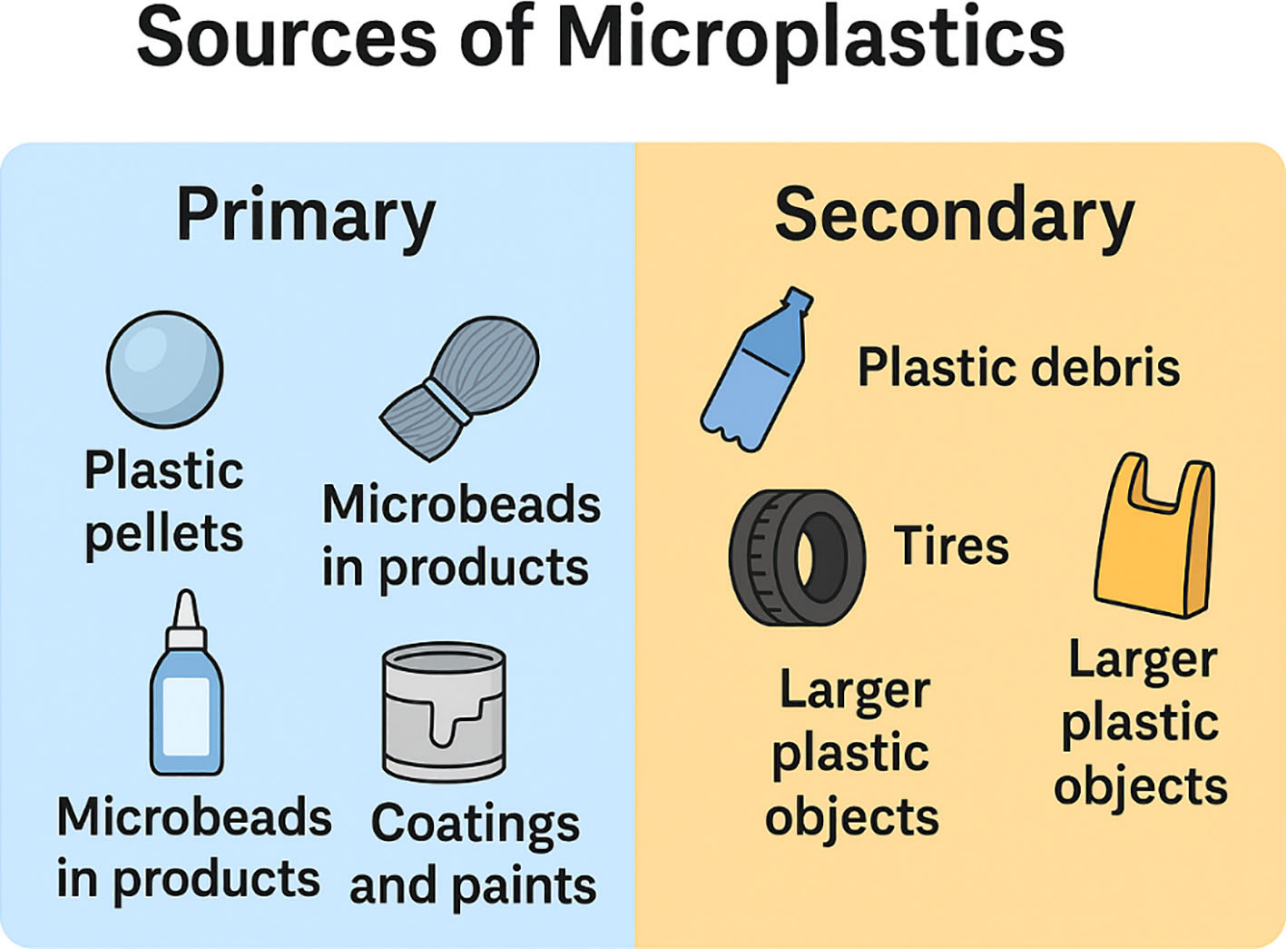
Sources of microplastics (Primary and Secondary).

The presence of microplastics in agricultural soils has become a major issue in agricultural soil due to the many causes that contribute to this contamination. Microplastic pollution in farmlands is mostly caused by agricultural operations (shown in **Figure 1**), including the usage of plastic mulch films, irrigation water, and biosolids application (Qi et al., 2019b). Research has shown that agricultural soils contain microplastics, with concentrations as high as 306 ± 360 particles/kg in croplands and tropical areas (Praveena et al., 2023; Corradini et al., 2020). According to Yang et al. (2021), biodegradable mulch films generate microplastic at a faster rate than oxodegradable and traditional polyethylene films. Furthermore, pesticides such as prothioconazole might enhance plastic breakdown and impact microplastics’ ability to adsorb heavy metals (Li et al., 2020a). Microplastics have complex effects on agricultural systems. In addition to influencing plant development, they may change the cheare considered ecologically relmical and physical characteristics of soil as well as the activities of microbes and enzymes (Xu et al., 2019). Microplastics have an effect on soil that increases evapotranspiration, organic carbon content, and microbial biomass, while lowering bulk density and microbial diversity (Zhang et al., 2022). Further study is needed to fully understand the long-term impacts of microplastic contamination in agriculture and to create appropriate mitigation methods. These results underscore the intricate connections between microplastics and the soil ecosystem (Tariq et al., 2024).

### 2.1 Types of microplastics and their sources

This review focuses on polyethylene (PE), polyvinyl chloride (PVC), and polypropylene (PP) microplastics due to their widespread presence in agricultural environments (**Table 2**). PE is extensively used in plastic mulching films, PVC in irrigation pipes and packaging materials, and PP in fertilizer bags and woven containers (Jia et al., 2022; Li et al., 2020b) (**Figure 1**). These three polymers are consistently reported among the most dominant types detected in agricultural soils globally (Guo et al., 2022). Additionally, they exhibit unique degradation pathways PE and PP primarily degrade via main chain random scission, whereas PVC follows branched chain scission which affects the release of by-products and their interaction with soil properties and microbial communities (Huang et al., 2018; Xu et al., 2023). Although other microplastics such as polystyrene (PS) and biodegradable PLA are also found, their agricultural occurrence and long-term behavior are less understood, thus justifying the emphasis on PE, PVC, and PP in this review (**Table 3**).

**Table 2:** Impact of different microplastics on soil and crop growth.

Microplastic type	Effect on soil	Effect on wheat growth	Citation
Polyethylene (PE)	Slows organic matter degradation, increases NH_3_ emissions	Reduces root and shoot growth, alters nutrient uptake	Qi et al., 2020
Polyvinyl Chloride (PVC)	Reduces NO_3_^-^ N levels, alters microbial communities	Most toxic, decreases leaf size, weakens photosynthesis	Liu et al., 2021
Polypropylene (PP)	Lowers ammonium-N levels, affects nitrification	Slows root development, reduces nutrient absorption	Zhang et al., 2022

**Table 3:** Comparative characteristics and impacts of major microplastic types in agricultural soils.

Polymer	Full name	Common agricultural sources	Persistence	Key chemical traits	Impacts on soil health/N cycling	Reference
PE	Polyethylene	Plastic mulch films, greenhouse covers, irrigation tubing	Very high	Inert, hydrophobic, nonpolar	Alters soil porosity and water retention; moderate disruption to microbial N-cycling	Qi et al., 2020; Wang et al., 2021
PP	Polypropylene	Fertilizer bags, ropes, seedling trays, packaging	High	Semi-crystalline, oxidation-resistant	Physically blocks root growth; affects microbial biomass and ammonification	Zhou et al., 2022; de Souza Machado et al., 2019
PVC	Polyvinyl chloride	Irrigation pipes, plastic sheets, low-cost packaging	Very high	Contains plasticizers, heavy metals, Cl content	Leaches toxic additives; alters soil pH, suppresses nitrifiers and denitrifiers	Lithner et al., 2011; Zhou et al., 2022
PS	Polystyrene	Food packaging, insulation, urban runoff	Moderate	Aromatic ring, brittle, low density	May inhibit plant root elongation; fewer studies in agri-soils	Rillig et al., 2019
PET	Polyethylene terephthalate	Clothing fibers, plastic bottles, irrigation runoff	Moderate–High	Polar, slow degradation	Low soil reactivity; limited microbial or chemical interference	Liu et al., 2021
PLA	Polylactic acid (bioplastic)	Biodegradable mulch films, compostable packaging	Low	Hydrolysable ester bonds; biodegradable	Initial toxicity possible; long-term effects minimal after degradation	Zhao et al., 2020

### 2.2 Characteristics of microplastics (size, type, concentration)

A nuanced understanding of microplastic behavior in soils requires examining their physical and chemical characteristics. These attributes such as particle size, polymer type, shape, and degradation pattern govern how microplastics interact with soil matrices, microbial communities, and nutrients (**Table 4**). Microplastics in agricultural environments often fall under the size category of <5 mm, but smaller fragments and nanoplastics increasingly dominate soil profiles due to progressive degradation (Liu et al., 2022a). Their morphological diversity, from fibers to pellets, along with compositional variability, strongly influences their environmental fate and ecological impact. Microplastics are plastic particles smaller than 5 mm, often classified into small microplastics (0.2–2 mm) and large microplastics (2–5 mm) (Collignon et al., 2013), with some as tiny as 4 μm (Liu et al., 2022a). They appear in different shapes, including fragments, fibers, films, and pellets, and are commonly composed of polyethylene, polypropylene, polystyrene, and polyethylene terephthalate (PET) (Nihei et al., 2023). Their colors vary, with white, blue, and yellow being frequently observed. The concentration of microplastics differs significantly across environments, ranging from 6.2 particles/100m² in marine surface waters to 91 ± 55 items/g dry weight in coastal sediments (Liu et al., 2022a). In river water, microplastics were detected at 99% of sampling stations (Nihei et al., 2023), while beach sediments showed variations from 0.27 to 1.35 particles/kg dry weight (Cocozza et al., 2024). Their accumulation in biological systems is evident, as mussels tend to over-represent modified-cellulose fibers but under-represent polyvinyl compared to the surrounding seawater and sediment (Scott et al., 2019). The relationship between microplastic concentration and size often follows a power-exponential equation, with smaller particles being more abundant (Liu et al., 2022a). Microplastic degradation in soils occurs through several mechanisms including photooxidation, thermal weathering, and microbial enzymatic action. These degradation pathways lead to the formation of nanoplastics, reactive oxygen species (ROS), and various chemical additives such as phthalates and bisphenol A (Sun et al., 2025; Xu et al., 2023). These by-products can penetrate microbial cell walls, disrupt enzyme activities, and alter soil pH and redox conditions, thereby interfering with nitrogen transformations like nitrification and denitrification (Qi et al., 2019a; Rillig et al., 2021). For example, biodegradable microplastics such as PLA release toxic intermediates during breakdown that have been linked to impaired microbial respiration and reduced compost maturity (Sun et al., 2020). Understanding these pathways is critical to evaluating the long-term impacts of microplastic contamination on soil health and microbial ecology.

**Table 4:** Microplastics concentration in agricultural soils.

Location	Microplastic concentration (particles/kg soil)	Citation
China Hetao Irrigation District	1,810 - 86,331	Liu et al., 2022b
Chile Central Valley	Detected in croplands but not in natural areas	Corradini et al., 2019
Cotton Fields with Long-term Film Mulching	3.20 ± 0.41×10^5^	Jia et al., 2022

They affect not only aquatic ecosystems but also terrestrial environments and air quality. The persistence and bio-accumulative nature of microplastics (Mbedzi et al., 2020) make them a long-term environmental concern. Furthermore, their ability to absorb and interact with other organic contaminants can increase their toxicity and complicate treatment efforts (Ali et al., 2023). Addressing these challenges requires immediate and collective action to restore balance in ecosystems and mitigate potential risks to human health (Marcharla et al., 2024).

## 3 Nitrogen cycle in agricultural ecosystems

**Figure 2:**
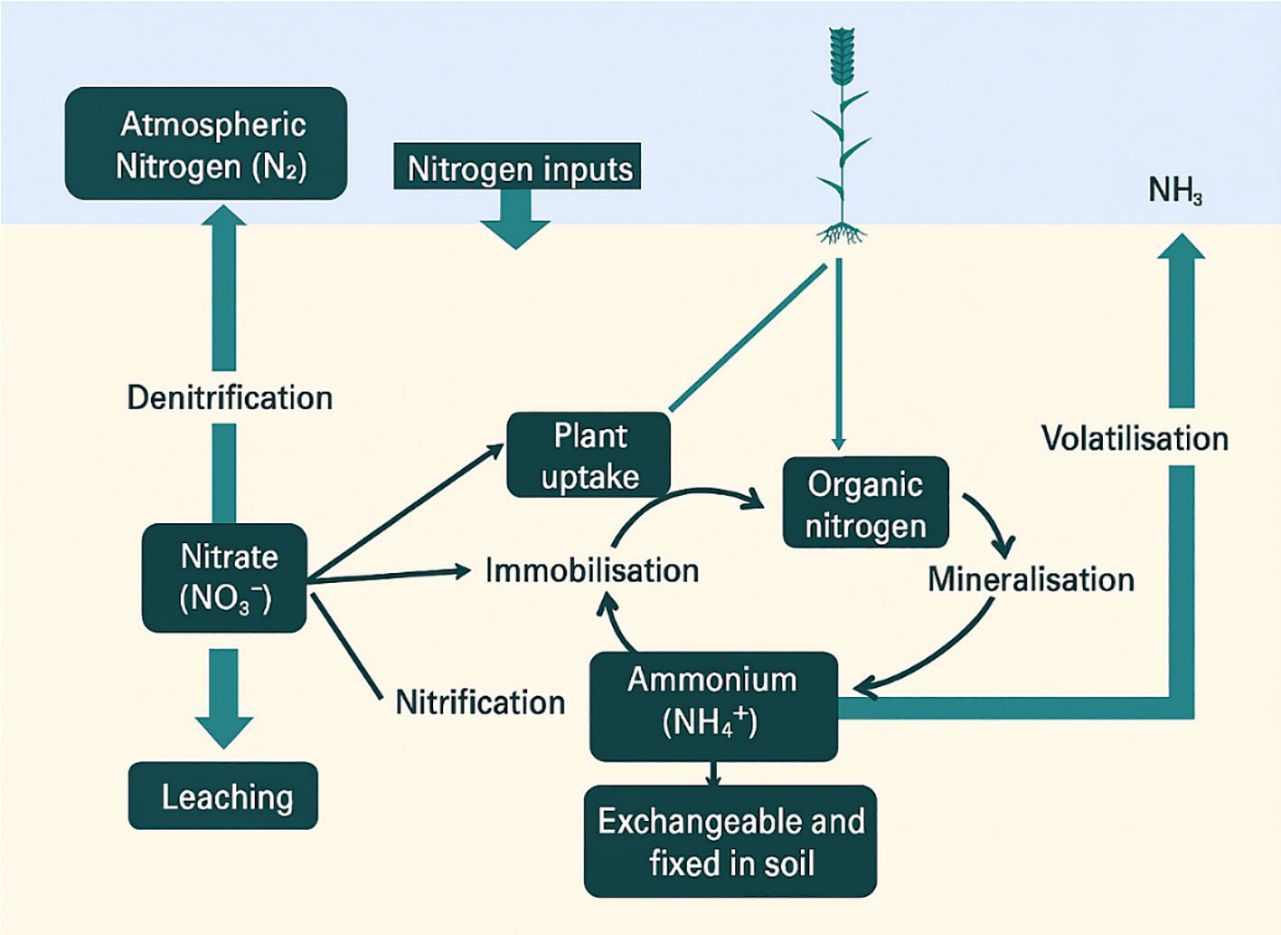
Nitrogen cycle in the soil (Nitrification, denitrification, immobilization, mineralization, ammonia volatilization).

**Figure 3:**
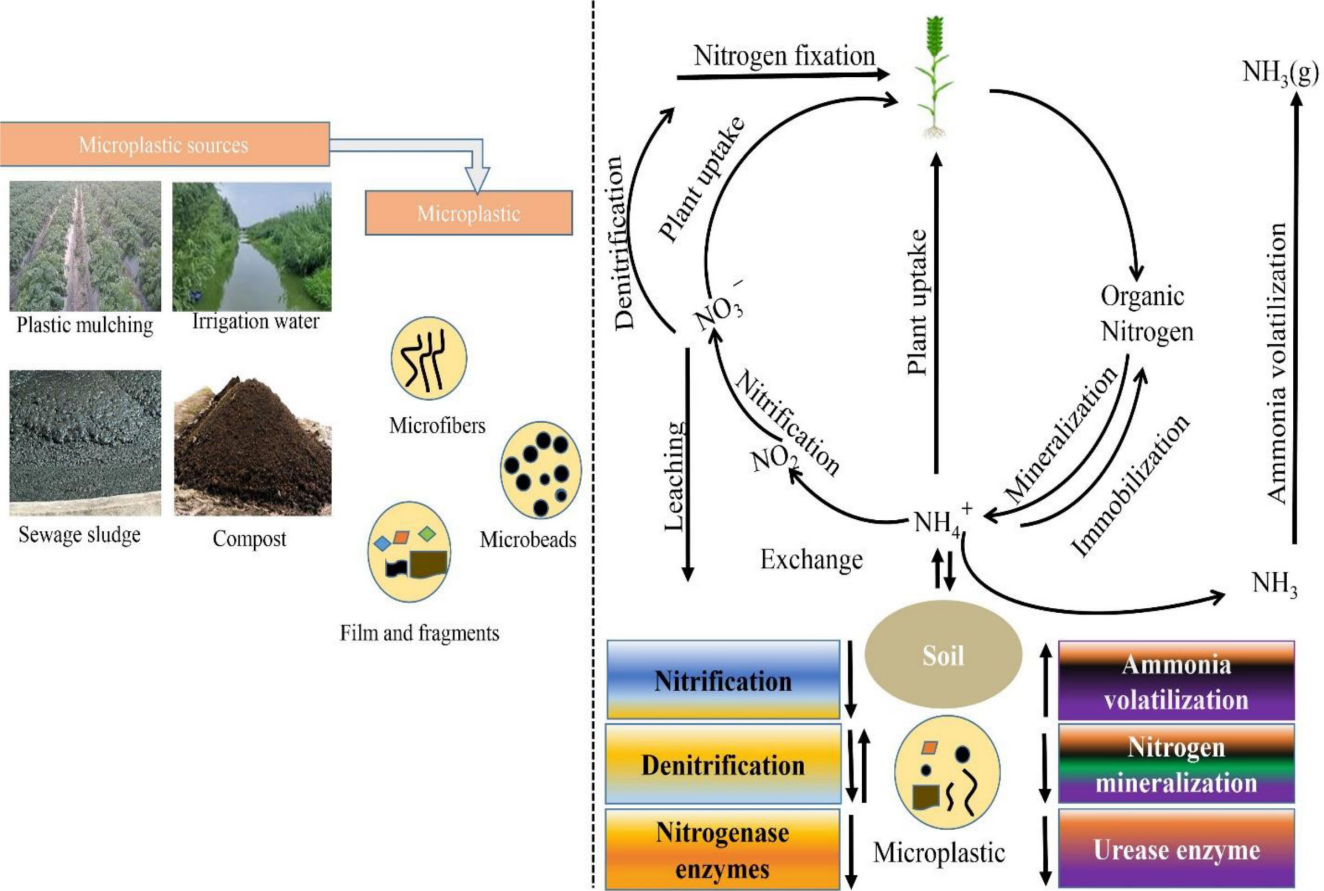
Sources of microplastics in agricultural soil and Impacts of microplastics on nitrogen cycling in the soil.

**Figure 4:**
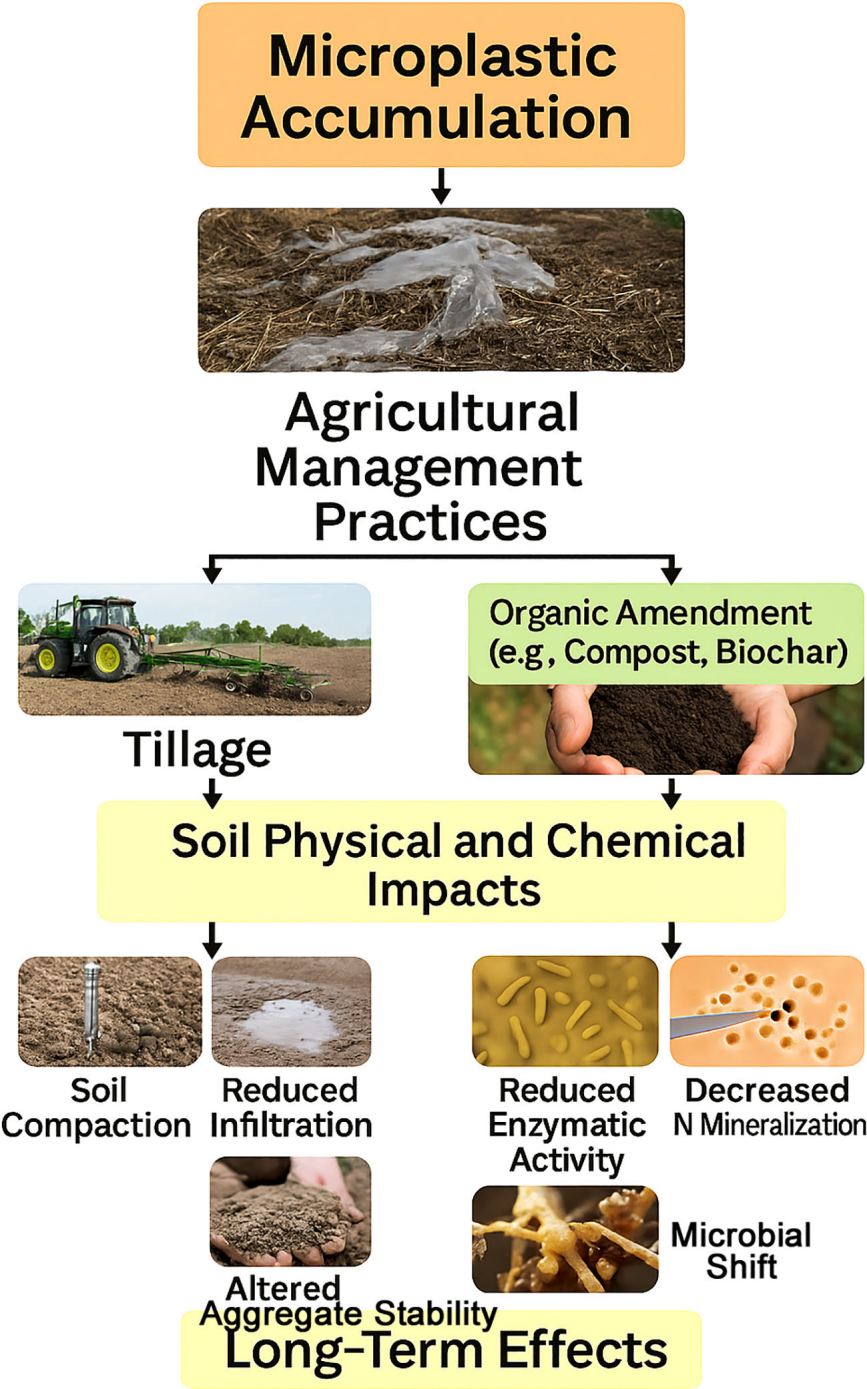
Microplastic accumulation flow chart.

**Figure 5:**
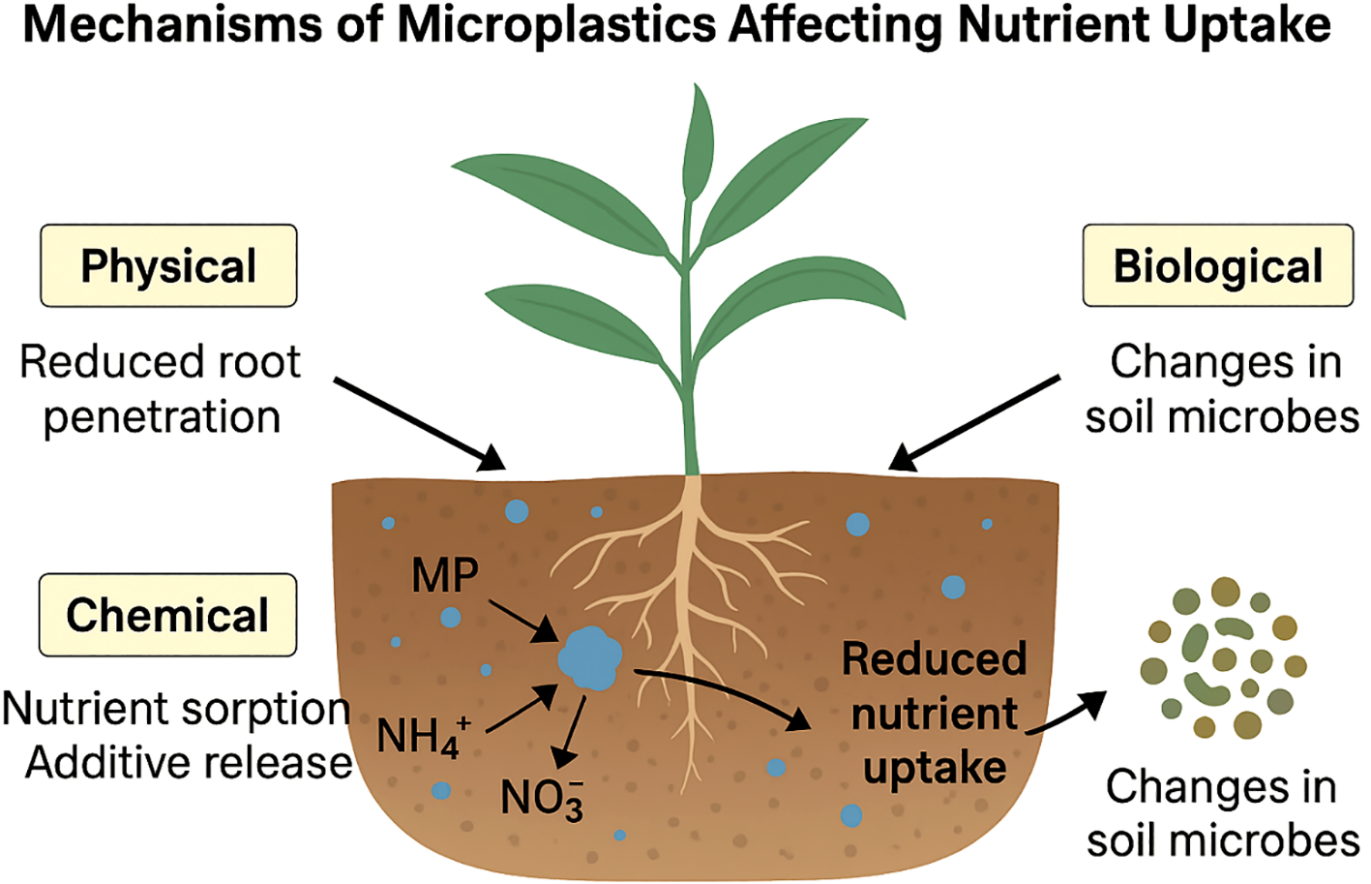
Mechanism of microplastic affecting nutrient uptake.

The nitrogen cycle is central to crop productivity, with microbial-mediated transformations governing the conversion of nitrogen between organic and inorganic forms (**Figure 2**). In agricultural ecosystems, managing these transformations efficiently is crucial for maintaining soil fertility and minimizing nitrogen losses. Processes such as nitrification, denitrification, and ammonia volatilization are influenced not only by soil conditions but also by emerging contaminants like microplastics (shown in **Figure 3**). Recent studies have shown that microplastics may alter microbial activity and enzyme function, thereby affecting nitrogen fluxes across soil systems (Wu et al., 2017; Chen et al., 2022). When it comes to agricultural ecosystems, soil microbes mediate critical changes in the nitrogen cycle (**Figure 3**). Plants have adapted to nitrogen scarcity by forming and attracting colonies of microbes that cycle nitrogen (Moreau et al., 2019). To keep soil fertility high and for sustained food production, these microbial interactions are crucial. Nutrient transformation in agricultural wetlands, such as rice paddies, is facilitated by periphytic biofilms, which consist of a broad array of microorganisms. They enhance nutrient use and decrease nonpoint source pollution by fixing nitrogen, activating occluded phosphorus, absorbing and storing bioavailable nitrogen, and so on (Wu et al., 2017). Periphytic biofilms improve nitrogen usage efficiency in rice fields by controlling the nitrogen cycle via the gradual release of excess nitrogen for reutilization (Chen et al., 2022). Agricultural ecosystems may benefit from better nitrogen management and preserved soil ecological function via the use of diverse cropping patterns. The abundance of nitrogen-cycling genes, such as nifH, nirS, nirK, and narG, is favorably impacted by these systems (Hao et al., 2022). Legumes may also be utilized to improve soils that are too salty by lowering the soil’s salinity and raising its nitrogen content by enriching nitrogen-fixing bacteria (Zheng et al., 2023). In addition to increasing soil quality, sustainable soil management strategies, including no-till farming, cover crop management, and manure application, may increase soil organic carbon storage, which in turn helps with sustainable food production (Komatsuzaki and Ohta, 2007) (**Figure 4**). Urea-based fertilizers vary in size, shape, and environmental impact (**Figure 5**). Common urea is typically found in granular (1–4 mm) or prilled (0.8–2 mm) forms, offering high solubility but rapid nitrogen release, making it susceptible to leaching, volatilization, and denitrification. In contrast, polymer-coated urea (PCU), generally 1–5 mm in size, is encapsulated in a synthetic polymer layer that controls nitrogen release, enhancing nutrient use efficiency (NUE) and reducing nitrogen loss. However, polymer coatings may degrade over time, contributing to microplastic contamination in soils and water bodies. As a more sustainable alternative, biochar-coated urea consists of granular particles (1–5 mm) covered with a biochar layer, which improves nitrogen retention, soil microbial activity, and carbon sequestration while avoiding plastic pollution. Another option is farmyard manure (FYM), a natural organic fertilizer with irregularly sized particles, composed of decomposed animal waste, urine, and bedding materials. FYM provides a slow-release nutrient source, enhances soil structure, and reduces reliance on synthetic fertilizers without the risk of microplastics pollution (**Table 5**). Given the environmental risks associated with synthetic polymer coatings, future research should focus on biodegradable alternatives and more rigorous monitoring of microplastic pollution originating from agricultural practices (Heiss and Fulweiler, 2016).

**Table 5:** Microplastic interaction with nitrogen fertilizers.

Nitrogen source	Microplastic interaction	Citation
Urea	Potential nitrogen adsorption, reduced efficiency	Wang et al., 2020
Farmyard Manure (FYM)	Possible microplastic contamination from animal waste	Zhang et al., 2021b
Biochar-coated Urea	Modifies soil structure, affects nitrogen release	Chen et al., 2022
Polymer-coated Urea	Potential alteration in nitrogen availability	Liu et al., 2023

### 3.1 Key processes in the nitrogen cycle (nitrogen uptake, nitrification, denitrification)

Nitrogen undergoes several important transformations throughout the nitrogen cycle. The main procedures, which are mostly performed by microbes such as bacteria, archaea, and certain specialized fungi, are nitrogen fixation, nitrification, nitrate assimilation, respiratory reduction of nitrate to ammonia, anammox, and denitrification (Martínez-Espinosa et al., 2011). Among the most crucial processes are nitrification and denitrification. Denitrification returns nitrate to atmospheric nitrogen, while nitrification converts ammonium to nitrite and finally nitrate. According to recent research (Heiss and Fulweiler, 2016), environmental factors may determine whether or not the two nitrification steps ammonium oxidation and nitrite oxidation are linked. Another important process in nitrogen removal by ecosystems is anaerobic ammonium oxidation, or anammox. In extreme instances, this process may account for as much as 67% of dinitrogen synthesis (Yang et al., 2012). The intricate series of reactions known as the nitrogen cycle controls the transformation of nitrogen into its many forms. Over the last hundred years, human activities have drastically changed the nitrogen cycle across the world, which has consequences for ecosystems and people’s health (Monib et al., 2024). To manage nitrogen in a variety of settings, including wastewater treatment, agricultural systems, and natural ecosystems, it is essential to understand these processes and how they interact.

### 3.2 Importance of nitrogen use efficiency in crop productivity

Nitrogen use efficiency (NUE) plays a crucial role in crop productivity and sustainable agriculture. It is essential for maximizing yields while minimizing environmental impacts associated with excessive nitrogen application. Improving NUE is a key objective in agroecosystem management, as it directly impacts grain yield, biomass production, and overall crop performance (Guo et al., 2016; Huggins and Pan, 2003). While nitrogen is vital for plant growth and crop productivity, NUE tends to decrease with increasing N supply, leading to resource waste (Wu et al., 2019). This highlights the importance of optimizing nitrogen management strategies to achieve a balance between productivity and efficiency. Furthermore, the effectiveness of NUE improvement techniques can vary depending on environmental and management factors, such as soil texture, irrigation systems, and nitrogen fertilizer rates (Abalos et al., 2014). Enhancing NUE is critical for developing sustainable agricultural practices that meet the growing demand for food while reducing environmental impacts. Strategies for improving NUE include site-specific nutrient management, integrated nitrogen management, and the use of advanced technologies such as remote sensing and crop simulation models (Jat et al., 2012). Additionally, understanding the molecular mechanisms underlying NUE and exploring genetic approaches for crop improvement offer promising avenues for future research and development in this field (Liu et al., 2015, 2022; Xu et al., 2012). Ammonia Volatilization in Soil Systems

Ammonia volatilization is a significant pathway of nitrogen loss from soil systems, influenced by various factors. Soil pH plays a crucial role, with higher pH leading to increased volatilization (Avnimelech and Laher, 1977). Moisture content also affects the process, as flooded soils generally exhibit higher ammonia losses compared to dry soils (Ventura and Yoshida, 1977; Vlek and Craswell, 1979). The type and application method of nitrogen fertilizers impact volatilization rates, with urea typically resulting in higher losses than ammonium sulfate (Vlek and Craswell, 1979). Some studies reveal contradictions in the effects of certain factors. While Schlesinger and Peterjohn (1991) suggest that competition by nitrifiers has little impact on ammonia volatilization, Mandal et al. (2015) indicate that nitrification can contribute to reduced volatilization when biochar is applied. Additionally, while most papers emphasize the importance of soil pH, Anaerobic digestion of pig slurry did not significantly alter ammonia losses despite changes in slurry properties (Chantigny et al., 2004). Ammonia volatilization in soil systems is a complex process influenced by multiple interacting factors. Management practices such as deep placement of fertilizers (Rao and Batra, 1983), use of slow-release fertilizers (Shan et al., 2015), and application of biochar (Mandal et al., 2015) can effectively reduce ammonia losses. Understanding these dynamics is crucial for developing strategies to mitigate nitrogen losses and improve fertilizer use efficiency in agricultural systems.

### 3.3 Mechanisms of ammonia volatilization

Nitrogen loss in agricultural and natural settings is mostly caused by ammonia volatilization. The primary regulator of the process is the rate of NH4+ mineralization from organic matter in the soil, which is accelerated by precipitation. Soil moisture, temperature, and pH are three of the many variables that affect the rate of ammonia volatilization. The increased concentration of hydroxyl ions in soils causes ammonia to volatilize more readily in environments where the pH is greater than 8. Volatilization rates often increase as temperature increases, suggesting that temperature is an important factor as well. A two-pronged impact of precipitation on ammonia volatilization is possible. It has dual purposes: increasing mineralization and volatilization rates, and decreasing volatilization via reducing the concentration of ammonium in the soil solution. The processes by which ammonia is vaporized are intricate and linked. The rate of volatilization is largely affected by the soil’s pH and the amount of NH4+ in the upper two centimeters of the soil (Chantigny et al., 2004). Another factor that may drastically lower volatilization rates is slurry with a surface crust. Applying biochar has the potential to decrease ammonia volatilization by as much as 70% via processes including nitrification and NH3 adsorption/immobilization (Mandal et al., 2015). To reduce nitrogen losses and increase nitrogen usage efficiency in agricultural systems, it is essential to understand these processes.

### 3.4 Factors influencing ammonia loss in agricultural fields

Factors about soil qualities, ambient circumstances, and fertilizer qualities impact ammonia volatilization from agricultural areas. Soil moisture, temperature, ammonium concentration, wind speed, and pH are important determinants (Chen et al., 2021). According to Shan et al. (2015), ammonia losses are more pronounced in soils with higher pH and ammonium levels. The rate of volatilization may be greatly affected by climatic variables such as wind speed and temperature; in general, higher temperatures result in larger losses (Sha et al., 2023). The relative importance of certain components might change under different circumstances. For example, in hot summer circumstances, the overall loss of ammonia was unaffected by slurry dry matter concentration, although its time course was (Thompson and Meisinger, 2002). Furthermore, components’ relative relevance may vary between crops grown in upland areas and those in paddy fields. Soil water content was critical for upland areas, while fertilizer type and ammonium concentration in ponding water were more important in paddy fields (Lee et al., 2024). There are a lot of moving parts in the complicated process of ammonia volatilization. The development of successful mitigation solutions relies on a thorough understanding of these factors. The use of slow-release fertilizers, quick inclusion of slurry, and subsurface fertilizer delivery are a few management strategies that may greatly decrease ammonia losses. Optimizing nitrogen usage efficiency while avoiding environmental consequences may be achieved by taking site-specific variables into account and selecting fertilizers appropriately.

## 4 Effects of microplastics on the nitrogen cycle

**Figure 6:**
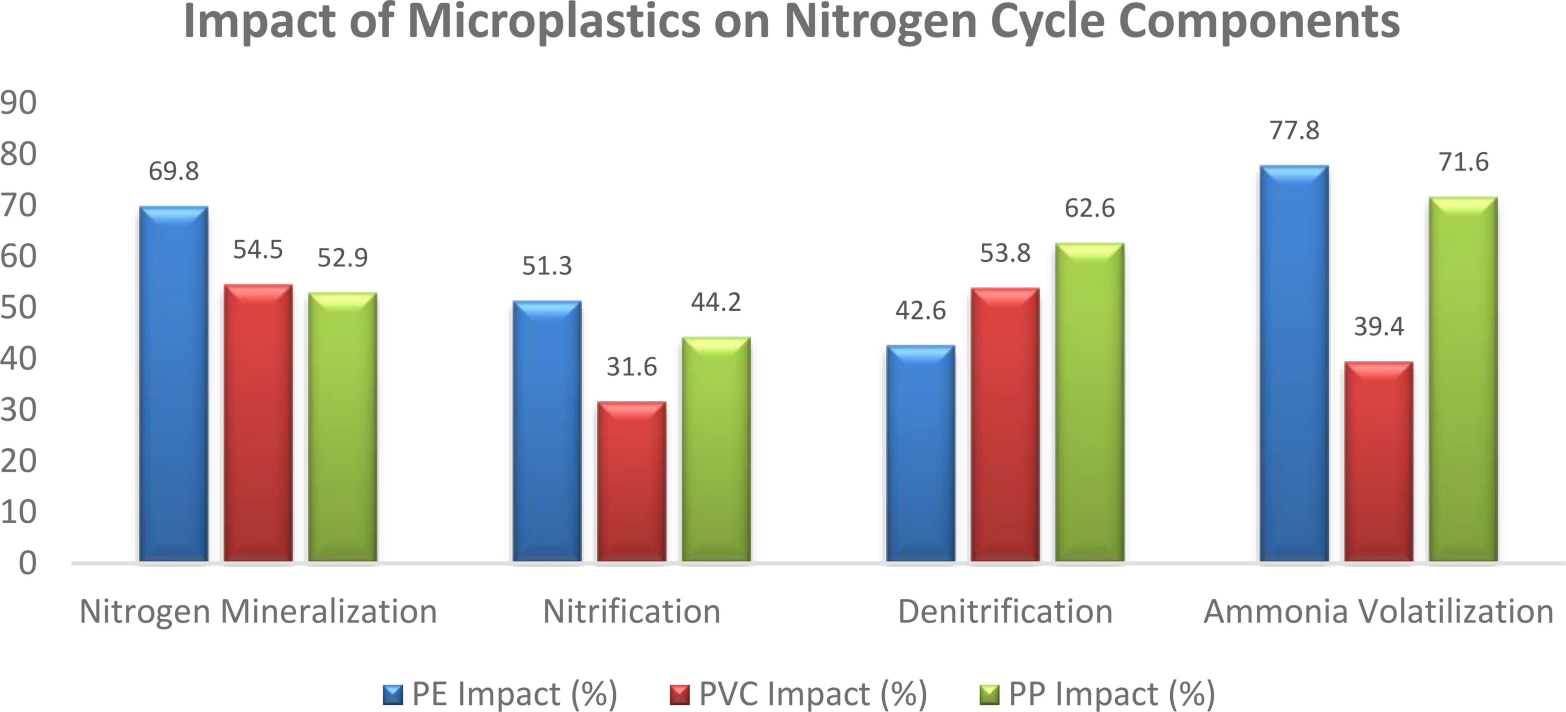
Impact of microplastics on nitrogen cycle components (Lu et al., 2021).

Some parts of the nitrogen cycle in composting and aquatic habitats have been seen to be affected by microplastics, especially polyethylene (PE), polyvinyl chloride (PVC), and polypropylene (PP). While polyvinyl chloride (PVC) microplastics slowed the decomposition of organic materials in compost, polyethylene (PE) increased emissions of methane (CH4) by 7.9-9.1% and ammonia (NH3) by 20.9-33.9%. But PVC reduced emissions of CH4 by 6.6% and NH3 by 30.4%. When compared to the control, PE and PVC both resulted in higher emissions of N2O (**Figure 6**). Furthermore, the nitrogen cycle was negatively affected by PE, PVC, and PHA microplastics, which decreased NO3- N concentrations and compost maturity. Microplastics’ effects on ammonia emissions and greenhouse gas emissions were shown to be source-dependent, indicating that various microplastics may affect nitrogen cycle mechanisms in different ways (Sun et al., 2020). Microplastics have been shown to influence aquatic biota, which may indirectly interfere with the nitrogen cycle. For example, Microplastic exposure in the gut of Caenorhabditis worms lowered calcium levels and raised glutathione S-transferase 4 enzyme expression, suggesting intestinal injury and oxidative stress (Lei et al., 2017). The decomposition of organic matter, emissions of greenhouse gases, and the nutritional content in compost are just a few areas where microplastics especially PE, PVC, and PP can have a substantial influence on the nitrogen cycle. The implications on ecosystem functioning and nutrient cycling in both aquatic and terrestrial ecosystems might be far-reaching.

**Figure 7:**
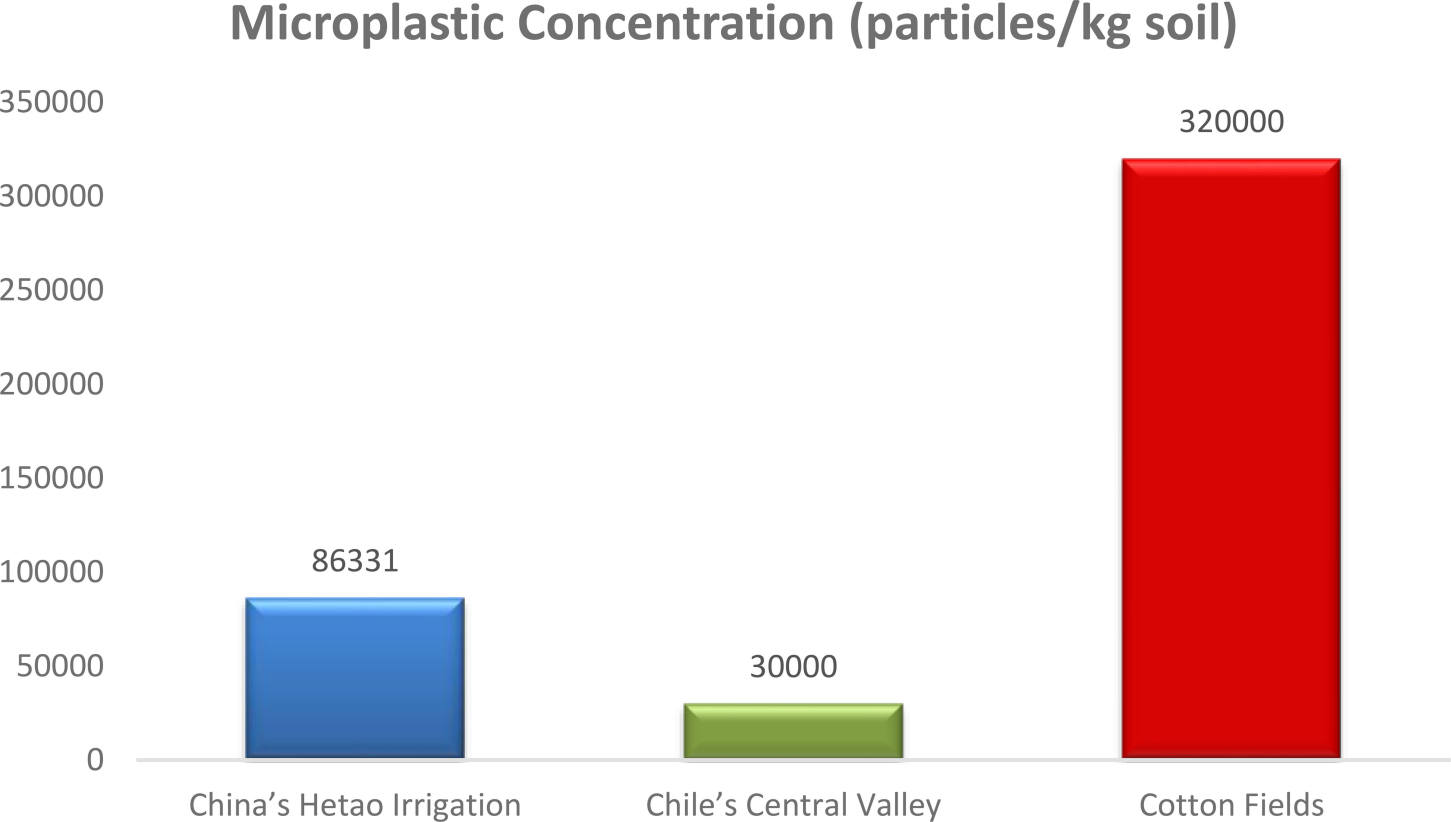
Microplastic concentrations (Liu et al., 2022b; Corradini et al., 2019; Jia et al., 2022).

The graphic below shows the many ways in which microplastics affect soil microbial populations and the nitrogen cycle (**Figure 7**). Dissolving microplastics into nanoplastics releases chemical additives and produces reactive oxygen species (ROS), which in turn inhibit functioning bacteria. Bacteria respond to this stress by becoming overwhelmed. According to Qi et al. (2019b) and Rillig et al. (2021), these stresses impair microbial cell walls, change the soil’s local environment, and produce anoxic conditions, which hinder nitrogen transformations, namely the conversion of nitrate (NO_3_^-^) to nitrogen gas (N_2_). Aeration and structural changes brought about by microplastics also affect redox states and microbial activity, leading to an increase in soil oxygen concentration. According to Li et al. (2020b), this alteration has the potential to throw off the equilibrium between ammonification and nitrification, which in turn impacts the amount of ammonium (NH_4_^+^) and nitrate that plants can absorb. Because microplastics change the composition, structure, and functional activities of microbial populations, a further important effect is the inhibition of microbial gene expression. Soil fertility and plant development may be adversely affected by this repression, which may limit microbial diversity and hamper nitrogen metabolism (de Souza MaChado et al., 2019). Nitrogen transformations are also impacted by microplastics, which change the activity of functioning enzymes, including urease, nitrate reductase, and nitrite reductase. Their inefficiency lowers soil production because it increases nitrogen losses (such as ammonia volatilization) and hinders nitrogen cycling (Liu et al., 2022c). Sustainable agriculture and environmental management must take into account the complicated and multidimensional impacts of microplastics on soil health, microbial dynamics, and nitrogen availability. These consequences are emphasized by these studies.

### 4.1 Impact on nitrogen mineralization, nitrification, and denitrification

Research on the effects of PE, PVC, and PP on nitrogen mineralization, nitrification, and denitrification in water settings has shown conflicting conclusions. Evidence suggests that polyethylene, vinyl chloride, and polypropylene microplastics may influence nitrogen cycling in activated sludge processes. Researchers discovered that these microplastics hindered the nitrification process by increasing the rate of ammonia oxidation and decreasing the rate of nitrite oxidation to a negligible degree. The denitrification process was improved by the incorporation of microplastics, especially PVC, at 5000 particles/L/L (particles per liter of water per liter of soil). In contrast, Three hour nitrification experiments with different amounts of microplastics did not substantially alter the total inorganic nitrogen (Li et al., 2019). The effects of various polymers on nitrogen-metabolizing microbes in wetland habitats have been the subject of conflicting research. The plastisphere microbial community structure and water nitrification and denitrification processes were impacted by PE, PS, and PVC, according to a 180-day exposure research in artificial wetlands. Wetland microbes that metabolize nitrogen were shown to be impacted by the degradation and age of these plastics (Zhang et al., 2023). Nitrogen cycling mechanisms may be affected by microplastics like PE, PVC, and PP, however, the exact nature of these impacts seems to rely on variables including concentration, duration of exposure, and environmental circumstances. It is worth mentioning that nitrous oxide (N2O) emissions during denitrification were considerably enhanced with high concentrations of PVC microplastic; in fact, 4.6 times greater emissions were produced with 10,000 particles/L of PVC compared to the control. These results emphasize the need to do more studies to determine the complete extent to which plastic pollution affects nitrogen cycling in terrestrial ecosystems over the long term.

### 4.2 Influence on nitrogen use efficiency and plant nitrogen uptake

Microplastics, which include polyethylene glycol, polypropylene, and other commonly used plastic polymers, make up a considerable amount of plastic trash (Huang et al., 2018). Degradation of PVC occurs by branched chain scission, while that of PE and PP mostly occurs via main chain random scission (Huang et al., 2018). A reactive force field molecular dynamics simulation was used to study the pyrolysis behavior of various plastics. The results demonstrated that the copolymerization of PVC/PE/PP mixes may influence the formation of gas phase products and the movement direction of free radicals (Xu et al., 2023). Research on the environmental presence of these polymers has focused on biosolids, which are treated sewage sludge. The majority of the plastics identified in bio solids were PE, which accounted for half of all plastics (Okoffo et al., 2020). PE had an average content of 2.2 mg/g dry weight. The accumulation of these polymers in soil settings might have an impact on soil qualities and plant development, according to this theory. Microplastics affect nitrogen uptake by disrupting multiple pathways. Physically, they alter root morphology by creating mechanical barriers in the rhizosphere, which limits root penetration and root hair development (Xu et al., 2023). Biologically, they impair microbial communities essential for nitrogen cycling, including nitrifiers, denitrifiers, and symbiotic nitrogen-fixers. Additionally, studies indicate that microplastics reduce the abundance of functional microbial genes such as *nifH* and *amoA*, thereby lowering nitrogen availability to plants (Guo et al., 2022). On a molecular level, microplastics can downregulate nitrate and ammonium transporter gene expression in plant roots. These disruptions are crop-specific: legumes and vegetables tend to exhibit greater physiological sensitivity due to their microbial dependencies and root system design. Moreover, environmental factors such as temperature, pH, and organic matter content can either buffer or exacerbate these effects.

## 5 Effects of microplastics on ammonia volatilization

Microplastics have varying effects on ammonia volatilization, depending on the type of plastic and environmental conditions. PE and PVC microplastics have been found to aggravate NH_3_ emissions during composting, with PE increasing emissions by 20.9-33.9% compared to control treatments without microplastics (Sun et al., 2020). However, PVC showed contradictory effects in different studies. While it decreased NH_3_ emissions by 30.4% in one composting experiment, PVC microplastics were observed to promote greenhouse gas emissions, including NH_3_, from farmland soil in another study. The effects of PP microplastics on ammonia volatilization were not explicitly discussed in the provided papers. However, PP was found to significantly promote emissions of other greenhouse gases like N_2_O, CO_2,_ and CH_4_ from soil (Chen et al., 2023), suggesting it may have similar effects on NH_3_ emissions. In summary, PE microplastics consistently increased NH_3_ emissions, while PVC showed variable effects depending on the environmental conditions. The impact of PP requires further investigation, but it likely promotes NH_3_ volatilization similar to other greenhouse gases. These findings highlight the complex interactions between different types of microplastics and nutrient cycling processes in soil and compost systems. The nitrogen cycle is incomplete without ammonia volatilization, the process by which organic matter or fertilizers convert ammonium (NH_4_^+^) into ammonia gas (NH_3_) and release it into the atmosphere. When nitrogen-rich fertilizers like urea or ammonium-based chemicals are added to agricultural soils, this process becomes very noticeable. Ammonia volatilization is accelerated by high soil pH, high temperatures, and low moisture levels, which results in significant nitrogen losses and decreased fertilizer efficiency (Wang et al., 2025). For example, if not mixed with soil, the nitrogen in urea, a popular fertilizer, can evaporate up to half of its original amount. As a result of these losses, farmers face increased input costs, a greater likelihood of environmental degradation, and a decrease in the amount of nitrogen available for crops (Kothari et al., 2024).

Targeted management measures are necessary to mitigate ammonia volatilization and provide a balanced nitrogen cycle. Fertilizer integration into the soil is a very efficient method since it decreases volatilization and surface exposure. A growing number of farmers are choosing to regulate the conversion of urea to ammonium and then to ammonia by using slow-release fertilizers and urease inhibitors. For example, research has shown that urease inhibitors may drastically improve crop yield and nitrogen usage efficiency by reducing ammonia volatilization by as much as 50%. Because it enhances nitrogen retention in soils and improves soil structure, biochar has also attracted attention as a soil supplement. This is because it lowers ammonia volatilization and adsorbed nitrogen compounds (Zhuang et al., 2025). Increasing soil organic matter via cover crops, compost, and crop rotation improved microbial activity and nitrogen cycling, which in turn reduced the need for synthetic fertilizers and reduced losses due to volatilization (Lakshmikanthan and Punithavathi, 2024).

Terrestrial and agricultural ecosystems rely on the nitrogen cycle, which converts nitrogen into forms that plants and animals can use on an ongoing basis. Amino acids, proteins, chlorophyll, and nucleic acids all rely on nitrogen, making it a fundamental macronutrient that plants cannot thrive without. According to Rahut et al. (2025), the nitrogen cycle is comprised of several interconnected processes that transform organic forms of nitrogen (N_2_) in the atmosphere into compounds that plants can easily absorb, such as ammonium (NH_4_^+^) and nitrate (NO_3_^-^). Soil fertility, plant production, and food security on a global scale are all affected by this cycle. There may be an adverse effect on agricultural systems and ecosystems as a whole when the nitrogen cycle is disrupted, whether by humans or natural causes.

### 5.1 Role of microplastics in altering soil pH and temperature

Agricultural soils may collect microplastics such as PE, PVC, and PP, with concentrations as high as 3.20 ± 0.41 x 105 particles/kg soil in places where farming has been going on for a long time (Jia et al., 2022). Soil microplastics can alter soil chemistry and characteristics via interactions with heavy metals and other contaminants. Microplastics’ sorption capacities for various chemicals are condition and polymer-specific. For example, according to Gao et al. (2019), heavy metals were more effectively absorbed by PVC and PP than by PE in both laboratory and field trials. Furthermore, organic chemicals such as sulfamethoxazole (SMX) may be adsorbed onto microplastics depending on the pH and salt levels (Guo et al., 2019). By changing the distribution of ions and organic molecules in the soil solution, these interactions can change the soil pH. Although it is not specifically mentioned in the given context how PE, PVC, and PP microplastics affect soil pH and temperature, their presence in soil and their interactions with other contaminants indicate that they may indirectly affect soil parameters. The precise function of these microplastics in changing soil temperature and pH requires more investigation.

### 5.2 Interaction with soil enzymes and microbial communities

The interaction of polyethylene (PE), polyvinyl chloride (PVC), and polypropylene (PP) with soil enzymes and microbial communities has significant implications for greenhouse gas emissions and environmental impact. Notably, PE was found to cause the most significant increase in greenhouse gas emissions as its pollution concentration increased (Chen et al., 2023). This suggests that these plastics can alter soil microbial activity and potentially contribute to climate warming. The degradation mechanisms of PE, PVC, and PP differ. PE and PP primarily degrade through main chain random scission, while PVC degrades mainly through branched chain scission (Huang et al., 2018). PE, PVC, and PP interact with soil enzymes and microbial communities in ways that can significantly impact greenhouse gas emissions and soil microbial ecology.

## 6 Microplastics and soil health

A major environmental problem, microplastic contamination in soil ecosystems impacts soil health and functionality. According to research, microplastics have the potential to change the chemical and physical properties of soil, which in turn affects hydraulic features, nutrient cycling, and soil stability (Boots et al., 2019). For example, according to Guo et al. (2022), microplastics hurt water retention capacity and saturated hydraulic conductivity of various soil textures, with clay soils being especially hard hit. Aspects such as soil texture, concentration, size, and form determine the impact of microplastics on soil ecosystems (Xu et al., 2019). Research has shown mixed results on the effects of microplastics on plant development, seed germination, and soil fauna (Boots et al., 2019; Habib et al., 2020). On the one hand, some microbes may be able to break down microplastics. A thorough understanding of the effects of microplastics on soil health and the wider ecosystem, as well as appropriate management techniques to address these consequences, is urgently required in light of the extensive prevalence of microplastics in agricultural soils (Qiu et al., 2022; Surendran et al., 2022) (**Figure 4**).

### 6.1 Effects on soil physical and chemical properties

Soil type, microplastics content, size, and form are among the variables that determine the degree to which microplastics alter soil physical and chemical characteristics. According to research, adding microplastics makes things more conductive under saturated conditions and increases the contact angle, but reduces the bulk density and water-holding capacity. Microplastics, when added to various soil textures, may lower saturated hydraulic conductivity; however, the impact is less pronounced for bigger particles (Guo et al., 2022). According to Yu et al. (2023b), soil qualities undergo slow but steady modifications, with noticeable changes happening only in areas with large concentrations of microplastics. Different types of soil and different features of microplastics might have different impacts on soil parameters. As an example, the physical qualities of soil might be more significantly impacted by polyester fibers than polypropylene granules, mainly because of the significant difference in form between the two. Furthermore, in soils that are more prone to erosion, microplastic fibers have been shown to decrease soil loss and sediment concentration (Ingraffia et al., 2022). On the other hand, microplastics may not significantly alter soil physical qualities in soils that are not severely contaminated with plastics at concentrations that are considered ecologically relevant (Yu et al., 2023a). Soil fertility and ecosystem functioning may be affected by microplastics, which change soil structure, water dynamics, and nutrient cycling (Kumar et al., 2022). Variables such as soil type, microplastics properties, and concentration all play a role in the complicated impacts.

### 6.2 Interaction with organic matter, nutrients, and microbial biomass

In both marine and terrestrial environments, microplastics have a major impact on organic materials, nutrients, and microbial communities. Soil and water quality, nutrient cycling, and the structure of microbial communities may all be profoundly affected by these interactions. Microplastics, dissolved organic debris, and hydrophobic microorganisms all tend to collect in the neustonic layer near the water-air interface in aquatic settings (Stabnikova et al., 2021). Biofilms may develop on the surfaces of microplastics at these concentrations, which may change the makeup and function of the microbial community (Arias-Andres et al., 2018). Due to microplastics’ strong attraction to DOM, which affects the composition, properties, and components of DOM in ecosystems, the interaction between the two is of special interest (Sun et al., 2022). Different types of plastic and different environmental factors may have different impacts on soil microbial communities and nutrient availability caused by microplastics. Soil hotspots were formed by the addition of polyhydroxyalkanoates (PHAs), which increased microbial biomass and activity and led to higher carbon and nutrient turnover (Zhou et al., 2021). Yan et al. (2020) highlighted the intricacy of these relationships by finding that soil bacterial populations and available phosphorus content were affected in mixed ways by polyvinyl chloride (PVC) microplastics at ecologically relevant quantities. Biogeochemical cycles and the ecological function of soil and water might be drastically changed by microplastics. They have the power to influence carbon dynamics in different ecosystems via their interactions with organic matter, nutrients, and microbial biomass, which in turn may establish new microbe niches, alter nutrient availability, and more. To completely comprehend the long-term effects of these interactions on the health and functioning of ecosystems, further study is necessary.

## 7 Impact on crop productivity

While wheat and rice are commonly studied due to their global importance, other crops show distinct responses to microplastic contamination (**Table 6**). For example, legumes like soybean and chickpea are particularly sensitive because of their reliance on nitrogen-fixing rhizobia, which are easily disrupted by microplastic-induced changes in microbial communities (Guo et al., 2022). Root vegetables such as carrot and radish, with direct soil contact, may experience altered root morphology and nutrient uptake due to physical obstruction or sorption of nutrients by microplastics. These differences are largely attributable to variations in root zone architecture, symbiotic microbial relationships, and crop-specific nitrogen demands. Therefore, understanding crop-specific vulnerabilities is essential for tailoring mitigation strategies.

**Table 6:** Comparative effects of microplastic exposure on selected major crop species.

Crop type	Scientific name	Root system & nitrogen strategy	Observed effects of microplastics	Possible explanation	Reference
Wheat	*Triticum aestivum*	Deep fibrous roots; nitrate uptake	Slight reduction in biomass and N uptake at moderate MP levels; delayed oxidative stress	Deep roots buffer physical disruption; soil depth dilutes MPs	Zhou et al., 2022
Rice	*Oryza sativa*	Shallow roots; ammonium preference	Reduced root length, chlorophyll content, and N assimilation; impaired microbial N cycling	Flooded conditions increase MP mobility; shallow roots more exposed	Qi et al., 2020
Maize (corn)	*Zea mays*	Deep roots; fast-growing; nitrate uptake	Decreased photosynthetic rate and biomass under MP exposure; limited root elongation	Rapid growth increases sensitivity to physical obstruction and oxidative stress	Meng et al., 2021
Soybean	*Glycine max*	Shallow taproots; symbiotic N fixation	Reduced nodule formation and nitrogenase activity; rhizosphere microbial disruption	MPs disrupt rhizobia populations and root exudate–microbe interactions	Lian et al., 2020
Tomato	*Solanum lycopersicum*	Shallow fibrous roots; nitrate uptake	Significant root damage, reduced chlorophyll and fruit yield under MP and MP+biochar treatments	High sensitivity due to shallow roots and low soil MP buffering	Qi et al., 2020
Lettuce	*Lactuca sativa*	Very shallow roots; nitrate uptake	Severe reduction in leaf area, root biomass, and nitrogen use efficiency	Small root system increases direct exposure; affected microbial community reduces nutrient availability	Zhou et al., 2022

### 7.1 Microplastic interaction with plants

Azeem et al. (2024) further emphasized that microplastics can enter the root system through both apoplastic and symplastic routes, depending on the particle’s surface charge and size. Their study indicated that nanoplastics are more likely to cross cellular membranes, leading to their accumulation in root and sometimes shoot tissues. This accumulation alters enzymatic activity, disturbs hormonal signaling, and induces oxidative stress responses such as elevated ROS production. Additionally, microplastics have been shown to adsorb agrochemicals, heavy metals, and persistent organic pollutants in the rhizosphere, which may enter plants along with the particles, exacerbating their toxic effects. These combined impacts highlight the complexity of microplastic fate in the soil–plant continuum.

### 7.2 Effects on plant growth, nitrogen uptake, and yield (i.e., Wheat)

More and more, agricultural soils include microplastics, which are little pieces of plastic that remain after bigger plastics break down. These microplastics may stunt plant development. Microplastics hinder root development and function, which is a big problem. According to research, microplastics may stunt a plant’s root and shoot development, making it less efficient in drawing soil nutrients and water. Wheat and rice, which rely on robust root systems to absorb vital minerals such as potassium, phosphorus, and nitrogen, may experience diminished plant vitality and decreased yields as a result. Inadequate nutrient absorption may make plants less robust and more vulnerable to drought and other forms of environmental stress. Microplastics have the potential to interfere with photosynthesis, the natural energy production process in plants. Microplastic exposure is associated with decreased photosynthetic efficiency and chlorophyll concentration, according to the research. Reduced chlorophyll concentrations hinder photosynthesis, a key process in plant energy production, since chlorophyll is so important in light absorption. According to Zhang (2020), this may cause wheat and rice to grow more slowly and produce less overall, which might limit their yield potential.

Changes to the nutritional makeup of plant tissues are another important consequence of microplastic pollution. Research has shown that plants may suffer from imbalances in nutrient concentrations, such as iron, due to microplastic exposure. Grain quality, nutritional value, and marketability may all take a hit when nutrients aren’t added properly to wheat and rice. The importance of nutrient-dense, high-yield crops to agricultural systems’ ability to provide food security makes this issue all the more pressing (Wright et al., 2013). Research investigating the long-term impact of microplastics on staple crops like rice and wheat is urgently needed due to their growing prevalence in agricultural soils. If we want to lessen the likelihood of threats to food production and security, we must learn how microplastics affect soil, plant roots, and crop physiology as a whole. According to D’Avignon et al. (2021), further research needs to focus on environmentally friendly farming methods that lessen the presence of microplastics in the soil without sacrificing its health or production. For example, wheat plants might be more susceptible to stress and disease if microplastics hinder the absorption of iron, a key element for plant metabolism and development. Also, the soil microbiome and other soil ecosystems might be affected by microplastics. Numerous microbes make up the soil microbiome, and they all work together to cycle nutrients, decompose organic materials, and keep the soil fertile. A decline in soil health may result from microplastics’ ability to upset microbial populations after they settle in the soil. Wheat relies on these microbes to break down nutrients and keep the soil in its proper structure, therefore, their alteration might stunt the plant’s development (Twardowska, 2020). For example, if nitrogen-fixing bacteria are negatively affected, soil nitrogen availability might be reduced, which would further restrict plant development.

Worries regarding microplastic buildup in the crop are heightened by their presence in the soil. Microplastics pose a threat to the food chain since they may be absorbed by wheat plants and ultimately wind up in the grains. The potential dangers that microplastics pose to both human and animal health make this a very pressing issue. Pesticides and heavy metals are only a few examples of the dangerous contaminants that microplastics may transport to the end user. Knowing how microplastics influence wheat development and food safety is crucial in light of this possible health concern (Galloway et al., 2017). There may be major ramifications for agricultural output and food security if microplastics harm crops like wheat. Wheat yields and quality might dip if root development is stunted, photosynthesis is impeded, nitrogen absorption is affected, and soil health is damaged. In addition, there has to be an immediate investigation into the long-term consequences of microplastic pollution on crops because of the possibility that they might infiltrate the food chain. To guarantee the safety of our food supply and reduce the impact of microplastics in agricultural systems, this study is crucial (Colzi et al., 2021; De Sá, 2018).

Microplastics have distinct effects on plant growth, according to studies conducted on zucchini plants (Cucurbita pepo L.) (Colzi et al., 2021). Among the materials that were evaluated, polyvinyl chloride (PVC) was shown to be the most poisonous. It had a substantial impact on leaf growth, photosynthesis, and plant iron content, weakening it even more. Both polypropylene (PP) and polyethylene (PE) stunted root and shoot development, but to a lesser extent; PE was the least detrimental of the three. These results provide further evidence that microplastics in soil might impede plant development and nutrient absorption, which in turn could impact harvest success. Soil quality and nutrient cycling are two other areas where microplastics affect plants. Composting was less successful using polyhydroxyalkanoate (PHA) microplastics because they sped up the degradation of organic materials, in contrast to polyethylene glycol (PEG) and polyvinyl chloride (PVC), which slowed it down (Sun et al., 2020). Furthermore, methane (CH4) and ammonia (NH3) emissions were raised by PE and PHA, which may have detrimental impacts on soil and air quality. PVC, on the other hand, reduced these emissions, although it still upset the soil’s nutritional balance. Nitrate (NO3- N) levels were lowered and compost maturation was delayed by all three kinds of microplastics, which may have limited the availability of nitrogen, a crucial nutrient for plant development.

The results of this research on composting procedures and zucchini have significant consequences on wheat harvests. Just like any other plant, wheat needs good soil, enough nutrients, and the right kind of root growth to thrive. Wheat fields contaminated with microplastics may experience stunted root and shoot development, decreased nitrogen absorption, and changed soil fertility. Wheat yields and grain quality might be negatively affected, which would have a knock-on effect on food production and farmers’ economic returns. Food safety concerns have also been raised by the possibility of microplastics transferring into wheat grains (Wright et al., 2013).

More study is needed to determine the precise impacts of microplastics on wheat and other staple crops, as they have been found in agricultural soils. Researchers need to find out how various soil types and amounts of plastic pollution affect plant vitality and productivity. Microplastics pose a threat to food production, but sustainable agricultural techniques, better waste management, and biodegradable materials may help reduce plastic pollution and its effects.

### 7.3 Influence of microplastics under different nitrogen sources (e.g., urea, FYM, biochar-coated urea, polymer-coated urea)

Soil microplastics, such as polyethylene (PE), polyvinyl chloride (PVC), and polypropylene (PP), interact with different types of nitrogen, which might affect soil chemistry and plant development. These microplastics are prevalent in agricultural soils. According to Gao et al. (2019), microplastics may change the dynamics of soil microbes and nutrient availability due to their capacity to absorb and transfer contaminants such as pharmaceuticals, heavy metals, and organic pollutants. One study revealed that cotton fields heavily mulched with plastic film had microplastic accumulations of up to 3.20 ± 0.41 × 10^5^ particles per kilogram of soil, demonstrating the seriousness of the problem in intensively managed farmlands (Jia et al., 2022). This indicates that agricultural lands are significantly contaminated with microplastics. This buildup is worrisome because it has the potential to impact the nitrogen cycle, namely by changing soil porosity, nutrient retention, and microbial activity. Soil characteristics, polymer type, and external environmental circumstances (such as moisture, temperature, and organic matter content) all play a role in how microplastics influence soil nitrogen dynamics. Soil pH, salinity, and the presence of co-existing organic contaminants might change microplastics’ sorption capacity, which in turn modulates their ability to interact with nitrogen sources (Guo et al., 2019). There is cause for worry about the sustainability of agricultural methods in the long run because these interactions may affect the bioavailability and effectiveness of various nitrogen fertilizers.

Nitrogen availability is a key component in wheat production systems that affects plant development, grain yield, and protein content. Wheat productivity might be severely affected if microplastics interfere with soil nitrogen retention in any way, whether it’s by binding nitrogen molecules or by changing microbial-mediated nitrogen transformation processes like nitrification and denitrification (Liu et al., 2021). Microplastics may alter the metabolic activity and nutrient conversion efficiencies of soil microorganisms, including nitrifying and denitrifying bacteria, by creating porous surfaces on which they can live (de Souza MaChado et al., 2018). The use of synthetic fertilizers like urea and controlled-release fertilizers like polymer-coated and biochar-coated urea to maximize nitrogen availability is common in wheat farming, making this a particularly pertinent issue. Soil nutrient imbalances may result from microplastics’ effects on the release dynamics of these fertilizers, which might be due to nitrogen absorption or changes in the microbial breakdown of organic nitrogen sources. Microplastics have been found to affect organic matter decomposition in different ways. One way is that biodegradable plastics, such as polyhydroxyalkanoates (PHA), can speed up decomposition. This could have indirect effects on nitrogen availability, according to research by Sun et al. (2020). Microplastics may restrict nitrogen absorption in wheat plants, which might influence crop development and output (Ren et al., 2021). This is because microplastics can diminish nitrate (NO^-3^N) concentrations and delay compost maturation.

Organic additives, such as biochar and farmyard manure (FYM), boost soil fertility by increasing organic matter content and microbial activity; synthetic fertilizers are also used in wheat cultivation. But how exactly these organic remedies interact with microplastics is still a mystery. Soil biochar and microplastics may alter soil structure and water retention capacity, which may affect nitrogen mineralization and absorption, according to studies (Huang et al., 2022). Wheat crops may have less efficient nitrogen usage because nitrogen-containing chemicals adsorb onto microplastic surfaces, making them less available for plant absorption. Due to animals’ ability to consume and excrete plastic particles, farmyard manure a byproduct of livestock production can also be a source of microplastics (Büks and Kaupenjohann, 2020). This makes us worry about the steady flow of microplastics onto farmland, where they may clog nutrient cycles for years to come.

Soil fertility and agricultural output aren’t the only things that might suffer as a result of microplastic pollution in wheat fields. Microplastics have the potential to build up in soil over time, changing the make-up of soil microbes and, in turn, impacting nitrogen cycle enzyme activity (Wang et al., 2022). There is cause for worry that toxins might be transferred into wheat plants and, eventually, the human food chain due to microplastics’ capacity to carry heavy metals and other environmental pollutants (Li et al., 2020a). The accumulation of microplastics-associated contaminants in wheat grains, a key ingredient in many people’s diets throughout the globe, raises concerns about food safety. There is an immediate need for more studies on the interactions between microplastics and various nitrogen fertilizers in areas where wheat is grown, since there is mounting evidence that microplastic contamination in agricultural soils might impact nitrogen cycling. Research should focus on studying the effects of various microplastics on nitrogen retention, microbial activity, and plant nutrient absorption in real-world settings.

Through interactions with several nitrogen sources, including urea, farmyard manure (FYM), biochar-coated urea, and polymer-coated urea, microplastics have the potential to greatly impact nitrogen cycling in agricultural soils. Soil properties, microplastics’ size, concentration, and kind all have a role in how these pollutants affect nitrogen availability, microbial activity, and soil structure. Fertilizers like urea are often used in farming, however tiny plastic particles may soak up nitrogen compounds, making it less effective since plants can’t absorb as much nitrogen (Wang et al., 2020). Microplastics, especially PE and PP, have been associated with altered nitrification rates and enhanced ammonia volatilization, which might cause soil nitrogen losses (Qi et al., 2020). Research has found microplastics in fertilizers made from manure, suggesting that organic fertilizer, Farmyard Manure (FYM), might be a culprit in this pollution (Zhang et al., 2021a). Nitrogen mineralization and transformations may be affected by microplastics in FYM and microbial populations; this, in turn, can affect the nutrient cycle and nitrogen efficiency (Huang et al., 2022). The goal of coating urea with biochar is to increase nitrogen retention and decrease leaching; nevertheless, microplastics have the potential to disrupt biochar’s adsorption capabilities. According to Chen et al. (2022), the nitrogen release dynamics might be changed by interactions between microplastics and biochar, which could lead to a decrease in the fertilizer’s efficiency. The nitrogen cycling process might be further altered if microplastic-induced changes in soil structure impact biochar’s nutrition and water retention capabilities. Soil microplastics may affect nitrogen diffusion and soil aggregation, which might affect the regulated release of nitrogen from polymer-coated urea. Research has shown that fertilizers coated with polymers may affect water retention and nutrient leaching patterns when they come into contact with microplastics (Liu et al., 2023). Additional study is needed to have a comprehensive understanding of the interactions and long-term consequences of microplastics and synthetic fertilizers in soil ecosystems. Microplastics have the power to change soil physical qualities, microbial nitrogen cycling, and nitrogen availability, as shown by their effects on various nitrogen sources (**Figure 5**). More study is needed to determine the combined effects of microplastic pollution of soils and the extensive use of nitrogen fertilizers so that sustainable agricultural practices may be implemented.

Emerging evidence suggests that the interaction between microplastics and fertilizer dynamics can vary significantly depending on soil type and climatic conditions (**Table 7**). For instance, in sandy soils, microplastics may enhance nutrient leaching by disrupting soil aggregation, whereas in clay-rich soils, they may reduce porosity and inhibit nitrogen mobility (Zhou et al., 2021). Soil pH also influences the sorption behavior of microplastics and their bound compounds, affecting nutrient availability. Climatic factors such as temperature and precipitation influence plastic degradation rates and microbial activity, which in turn mediate nitrogen transformations (Li et al., 2022). In arid regions, microplastics tend to persist longer due to slower degradation, while in humid zones they may undergo more fragmentation, increasing their surface reactivity. Therefore, regional agro-environmental conditions must be accounted for when assessing microplastic-fertilizer interactions and associated emission risks.

**Table 7:** Context-dependent interactions between microplastics and fertilizers across soil types and climates.

Condition	MP–fertilizer interaction dynamics	Agronomic implications	Supporting references
Sandy Soils	- MPs move freely, low adsorption - High risk of nutrient leaching with urea or nitrate	Reduced nitrogen use efficiency (NUE); nitrate contamination risk	Liu et al., 2022b; Xie et al., 2023
Clay Soils	- MPs accumulate near surface - Restricted aeration increases NH_3_ volatilization - Greater MP–N immobilization	Microbial inhibition and localized nitrogen loss near the rhizosphere	de Souza MaChado et al., 2019
Organic-Rich Soils	- Organic matter may buffer MP toxicity - Higher microbial resilience	Improved N cycling under MP stress; but risk of long-term additive accumulation	Zhou et al., 2022
Acidic Soils (pH < 6)	- Additive leaching from PVC intensified - MPs may enhance nutrient immobilization or disrupt microbial activity	Reduced ammonification and nitrification; lower NUE	Lithner et al., 2011; Wang et al., 2021
Alkaline Soils (pH > 7)	- Less MP degradation - Higher NH_3_ volatilization if urea used - Stronger biochar–MP–N synergy possible	Suitable for biochar-coated urea strategies; monitor NH_3_ emissions	Zhang et al., 2022
Arid Climates	- Low microbial activity slows MP and fertilizer degradation - MPs accumulate at surface	Delayed nutrient release; poor synchronization with crop N demand	Xie et al., 2023
Humid Climates	- MPs affect water retention - Enhanced N leaching with rainfall - Potential MP–urea hydrolysis acceleration	High risk of nutrient loss; environmental pollution if not mitigated	Liu et al., 2022c; Qi et al., 2020
Temperate Climates	- Moderate MP degradation - Strong seasonal influence on microbial–fertilizer interactions	Need for seasonal fertilizer adjustments; biochar blends effective	Wang et al., 2021

## 8 Biochar as a remediation strategy

As a remediation method, biochar has shown encouraging results in reducing the detrimental impact of microplastics on wheat crops. Research shows that biochar may improve crop yields while simultaneously decreasing the negative effects of microplastics on soil qualities and plant development. Biochar, especially biochar made from sawdust or dung, may accelerate the oxidation and breakdown of microplastics when added to soil that already contains these contaminants (Zhou et al., 2023). Soil microplastic concentrations may be reduced by this breakdown process, which may lessen the effect of microplastics on wheat plants. Additionally, research has shown that biochar may control the growth of some bacteria, which in turn reduces emissions of greenhouse gases and speeds up the breakdown of microplastics (**Table 8**). Although biochar has a positive impact on soil qualities and plant development in general, the extent to which it does so depends on the environmental factors and pollutants at play. In cadmium-contaminated soils, for example, Applying biochar surprisingly enhanced the availability and absorption of cadmium by wheat plants (Miao et al., 2023). Nevertheless, the impact was not directly linked to microplastics but rather to changes in soil pH and organic matter concentration. To mitigate the impact of microplastics on wheat harvests, biochar has shown promise as a remediation technique. It may enhance soil characteristics and increase microplastics breakdown, which can help reduce the detrimental effects of these pollutants. Soil type, contamination levels, and biochar characteristics are a few of the variables that may affect biochar’s efficacy. To find the best ways to apply biochar to different soil-microplastic-crop systems, further study is required.

**Table 8:** Biochar characteristics and their effectiveness in mitigating microplastic-induced soil impacts.

Biochar attribute	Options/Ranges	Effect on microplastic impact mitigation	Remarks/Recommendations
Feedstock Type	- Woody biomass (pine, oak, bamboo) - Manure or crop residues	- Woody: High surface area, better MP sorption - Manure-based: Enhances microbial activity but less stable structure	Prefer woody feedstock for MP binding; manure for microbial support
Pyrolysis Temperature	- Low (300–400 °C) - High (500–700 °C)	- Low: More functional groups, enhances microbial recovery - High: High surface area, greater MP and toxin adsorption	Use moderate to high temp (450–600 °C) for optimal balance
Surface Functional Groups	- Rich in COOH, OH, phenolics	Improves cation exchange and binding of MP additives (e.g., phthalates, heavy metals)	Target oxidized or functionalized biochar
pH Level	- Neutral to alkaline (pH 7–9)	Buffers soil acidity induced by MP breakdown products; supports nitrifier populations	Avoid extremely acidic or highly alkaline biochar
Post-processing	- Aged biochar - Biochar–compost blends - Biochar-coated urea	- Aging enhances MP adsorption via biofilms - Blends improve microbial resilience - Coated urea reduces NH_3_ loss and N leaching	Recommend blended or functionalized biochar for MP soils
Stability (Aromaticity)	- High carbon stability (H/C ratio < 0.3)	Ensures long-term MP immobilization and structural resilience in soil	Use highly aromatic, stable biochars for lasting remediation

Biochar has been widely recommended as a remediation strategy due to its porous structure, which enhances sorption of microplastics and reduces nitrogen losses (Chen et al., 2021). However, its application is not without risks. Certain biochars may alter soil pH unfavorably, especially in already alkaline soils, potentially affecting crop growth and microbial balance. Moreover, biochars derived from sewage sludge or industrial waste may contain heavy metals, introducing new contaminants (Liu et al., 2022c). The efficacy of biochar also varies significantly depending on feedstock type, pyrolysis temperature, and field conditions. From an economic standpoint, smallholder farmers may find biochar production or purchase cost-prohibitive without subsidies or incentives. These factors must be critically evaluated before recommending large-scale application.

The effectiveness of biochar in mitigating microplastic-related soil risks depends heavily on its physicochemical properties. Biochars produced from woody biomass at higher pyrolysis temperatures (above 500 °C) typically have greater surface area, aromaticity, and stability, which enhances their sorption of organic pollutants and polymer fragments (Tang et al., 2020). These high-temperature biochars also exhibit stronger resistance to microbial degradation, allowing for long-term benefits in soil systems. In contrast, low-temperature biochars tend to have more labile organic matter but lower adsorption capacity. Additionally, the feedstock origin influences nutrient content and porosity biochars from crop residues may retain more nitrogen, while those from manure sources may contain higher ash and mineral contents (Zhang et al., 2022). Tailoring biochar properties to specific soil and contamination conditions is thus crucial for optimizing its role in remediating microplastic pollution.

Despite its benefits, large-scale application of biochar raises several ecological concerns. Long-term use may alter microbial community structure by favoring specific taxa adapted to high carbon environments, potentially reducing microbial diversity. Changes in microbial composition can influence key soil processes such as nitrification and denitrification, thereby modifying nitrogen cycling. Additionally, excessive biochar application may immobilize nutrients like nitrogen and phosphorus or alter soil pH, leading to nutrient imbalances in certain soils (Sun et al., 2020). Regarding greenhouse gas emissions, some studies have reported reductions in N_2_O emissions, while others have observed no significant change or even increases under specific conditions, depending on feedstock and application rates. These mixed outcomes emphasize the importance of site-specific evaluations before recommending large-scale adoption of biochar as a remediation strategy.

Biochar, a carbon-rich material derived from pyrolysis of biomass, has emerged as a promising tool for mitigating MP contamination in soils. Its efficacy is attributed to multiple physicochemical properties, including high surface area, porosity, and diverse functional groups (–OH, –COOH) that facilitate interactions with MPs (Duan et al., 2025; Wu et al., 2023).

### 8.1 Mechanisms of MP immobilization

### MP Absorption on biochar surfaces occurs through:

- π–π interactions between aromatic structures in biochar and polymer backbones of MPs.
- Hydrophobic partitioning favoring adsorption of nonpolar MPs within biochar micropores.
- Electrostatic interactions mediated by biochar surface charges, particularly at different pH levels.
- Hydrogen bonding and surface functional group interactions that stabilize MPs on biochar matrices (Tan et al., 2015).

### Recovery Efficiencies and Long-Term Stability

Studies report MP removal efficiencies of 45–78% when soils are amended with biochar derived from agricultural residues (Duan et al., 2025). However, the long-term stability of immobilized MPs remains uncertain, with potential desorption under changing environmental conditions (e.g., pH shifts, microbial degradation).

### Ecological Implications

While biochar can improve soil structure and nutrient retention, concerns persist about its potential to act as a vector for co-contaminants or alter soil microbial communities (Su et al., 2024; Mota et al., 2025). Future work should explore trade-offs between remediation efficacy and unintended ecological consequences.

### 8.2 Potential of biochar to mitigate microplastics effects

Microplastics (MPs), including polyethylene (PE), polyvinyl chloride (PVC), and polypropylene (PP), are widely present in soil environments, raising concerns about their long-term environmental and agricultural impacts. Biochar has shown significant potential in mitigating MPs pollution by enhancing their degradation and reducing their negative effects on soil properties, plant growth, and microbial activity. Studies have demonstrated that biochar can promote the oxidation and breakdown of MPs, effectively reducing their concentration in composting processes (Zhou et al., 2023). This makes biochar a valuable amendment for remediating MPs contaminated agricultural soils, particularly at lower contamination levels, where it helps restore soil health and improve plant growth (Elbasiouny et al., 2023). Beyond direct mitigation, biochar can enhance the remediation of other contaminants in MPs-polluted environments. Research has shown that the combination of biochar and polyethylene plastic fragments accelerated the removal of polycyclic aromatic hydrocarbons (PAHs) and phthalate esters (PAEs) from contaminated soil (Ren et al., 2021). This effect is attributed to the high sorption capacity of both biochar and MPs, as well as the increased abundance of PAH and PAE-degrading microorganisms. By improving microbial degradation and adsorption processes, biochar serves as a multifunctional soil amendment that not only reduces MPs pollution but also limits the emission of greenhouse gases in contaminated soils (Wang et al., 2023). Despite these benefits, the long-term interactions between biochar and MPs in soil environments require further study. Some research suggests that biochar and MPs may form heterogeneous aggregates, which could alter their sorption behavior for organic contaminants (Yao et al., 2023). Understanding these interactions is essential for optimizing biochar application strategies across different environmental conditions. Future research should focus on determining the most effective biochar types, application rates, and environmental conditions that enhance MPs degradation and removal, ensuring its effectiveness as a sustainable solution for MPs remediation in soil and water systems.

### 8.3 Biochar’s role in improving nitrogen use efficiency and reducing ammonia volatilization

Biochar has shown significant potential in improving nitrogen use efficiency (NUE) and reducing ammonia volatilization in agricultural systems. Multiple studies have demonstrated that biochar application can decrease ammonia volatilization by up to 70% (Mandal et al., 2015). This reduction is attributed to various mechanisms, including ammonia adsorption/immobilization and enhanced nitrification (Cai et al., 2022). Biochar also improves NUE by increasing nitrogen uptake in crops, with studies reporting up to a 76.11% increase in wheat N uptake. The effects of biochar on ammonia volatilization can vary depending on application rates and soil conditions. While low rates of aged biochar decreased NH_3_ volatilization, high rates and fresh biochar reapplication increased it (Dong et al., 2019). This contradiction highlights the importance of optimizing biochar application strategies. Additionally, biochar-coated urea (BCU) has shown promise in reducing nitrogen loss, primarily through reduced nitrate leaching, although it may enhance ammonia volatilization due to increased soil NH_4_^+^N concentration and pH. Biochar demonstrates significant potential for improving NUE and reducing ammonia volatilization in agricultural systems. However, its effectiveness depends on various factors, including application rate, soil conditions, and biochar properties. Combining biochar with other management practices, such as plastic film mulching, can further enhance its benefits (Liu et al., 2022c). Future research should focus on optimizing biochar application strategies to maximize its positive effects on nitrogen management in agriculture.

## 9 Challenges and knowledge gaps

Despite growing research on microplastic contamination in agricultural soils, significant gaps remain in understanding its full impact on soil health, nitrogen cycling, and crop productivity. Many studies focus on laboratory experiments or short-term trials, limiting the ability to assess long-term consequences under field conditions. The complexity of microplastics’ interactions with soil nutrients, microbial communities, and crop growth necessitates more comprehensive, real-world investigations. Current research also lacks consistency in methodology, with variations in microplastic concentrations, particle sizes, exposure durations, and sampling protocols making it difficult to compare findings across studies. To improve comparability, researchers have proposed standard extraction techniques such as density separation using zinc chloride (ZnCl_2_) solutions and sieving for particle recovery (Guo et al., 2022). Identification methods like Fourier-transform infrared spectroscopy (FTIR), Raman spectroscopy, and scanning electron microscopy (SEM) are increasingly applied for polymer type classification (Liu et al., 2022b). Furthermore, organizations like ISO (e.g., ISO/TC61/SC14) and FAO have initiated efforts to establish unified protocols for detecting microplastics in agricultural soils. Adoption of such approaches will significantly improve the reliability of microplastic impact studies across diverse agro-ecological contexts.

Over extended periods, microplastic accumulation can result in physical and biological alterations to soil systems. Long-term presence of polymers like polyethylene and PVC has been linked to reduced soil porosity, disrupted microbial community structure, and impaired enzymatic activity critical for nitrogen cycling. Agricultural practices such as intensive tillage may accelerate vertical movement of microplastics, while no-till systems can lead to their surface accumulation. Similarly, manure application, compost use, and plastic mulching may contribute to additive effects of plastic deposition over time. These cumulative impacts are not yet fully understood due to the absence of long-term field experiments. Therefore, establishing longitudinal studies under contrasting management regimes is essential for understanding how persistent microplastics alter soil health, crop productivity, and greenhouse gas emissions across different agroecosystems.

### 9.1 Policy implications and sustainable management recommendations

Based on the scientific findings discussed in this review, several targeted policy actions are necessary to mitigate microplastic contamination in agricultural systems (**Table 9**). First, the use of polyethylene-based mulching films should be phased out or restricted, encouraging the adoption of biodegradable alternatives that have been field-tested for environmental compatibility (Avinash et al., 2023). Compost and biosolid products must include mandatory labeling of microplastic content to ensure transparency and accountability, as these materials are often applied to soil without awareness of their plastic load. National monitoring programs should be initiated to routinely assess microplastic concentrations in agricultural soils, utilizing standardized protocols such as density separation and polymer identification through FTIR or Raman spectroscopy (Liu et al., 2022c). Additionally, biochar application should only be promoted in areas where field-level validation confirms its agronomic and ecological benefits, taking into account the variability of feedstock sources and production conditions. Finally, successful implementation of these strategies depends on collaboration among environmental authorities, agricultural institutions, and local farming communities, fostering a shared approach to sustainable land management and soil health protection.

**Table 9:** Science-to-policy connections for managing microplastics in agriculture.

Scientific finding	Policy implication/actionable recommendation
MPs disrupt nitrogen cycling and increase emissions	Integrate MP risk into national nutrient management and GHG reduction strategies
Biochar can mitigate MP effects but needs standardization	Develop biochar quality certification schemes and field-use guidelines
PE, PVC, and PP are dominant MPs in farm soils	Regulate plastic use in agriculture; incentivize biodegradable mulching and packaging
Microbial communities are altered by MP exposure	Revise soil health monitoring programs to include microbial indicators in MP-affected zones
Lack of standardized testing protocols	Mandate ISO-compliant MP detection in soil and agri-input regulation (e.g., compost)
Legumes and shallow-rooted crops are more vulnerable	Promote crop-specific risk assessments and adaptive agronomic recommendations

### 9.2 Limitations in current research

One of the major limitations in existing research is the predominance of controlled laboratory experiments, which do not accurately replicate real-world agricultural conditions. Many studies rely on artificially introduced microplastics, often with uniform size and shape, whereas field-derived microplastics are more diverse in composition and degradation state. Additionally, most studies assess the effects of microplastics over short periods, typically weeks or months, rather than examining their cumulative impact over years of agricultural use. This limits our understanding of how microplastics persist in soils, interact with fertilizers, and influence long-term soil fertility and plant health. Another limitation is the lack of standardized methods for detecting and quantifying microplastics in agricultural soils, leading to inconsistent reporting and difficulty in comparing findings across different studies. Furthermore, while some research has investigated microplastic accumulation in plant tissues, there is limited information on whether these particles can be transferred to edible parts of crops, such as wheat grains, posing potential risks to food safety. Additionally, existing studies often focus on individual nitrogen sources rather than examining how microplastics interact with multiple fertilizers under different soil conditions, which would provide a more comprehensive understanding of their impact on nitrogen use efficiency.

Another key limitation in microplastic research is the absence of harmonized and standardized methodologies, which hinders data comparison across studies and ecosystems. Currently, researchers employ varied approaches for microplastic sampling, extraction (e.g., density separation), and polymer identification (e.g., FTIR, Raman spectroscopy), leading to inconsistencies in reported concentrations and types (Rillig et al., 2021). International efforts, such as those proposed by the Global Soil Partnership and ISO, are underway to create uniform guidelines for soil microplastic analysis. For instance, Li et al. (2022) highlighted the importance of defining size thresholds, sampling depth, and chemical pretreatment steps to enhance reproducibility (**Table 10**). Until such frameworks are universally adopted, the interpretation of microplastic impacts on soil and plant systems will remain uncertain and fragmented.

**Table 10:** Comparative summary of biochar advantages vs. limitations in microplastic-contaminated soils.

Aspect	Advantages	Limitations/risks
Soil Health	Improves soil structure, porosity, and water-holding capacity	May alter pH and redox potential; can suppress beneficial microbial populations at high doses
Nutrient Retention	Reduces nitrogen leaching and ammonia volatilization; enhances cation exchange capacity	Variable nutrient sorption depending on biochar type; potential immobilization of plant-available nutrients
Microbial Interactions	Supports microbial habitat and activity in aged biochar	May cause microbial shifts or inhibition in freshly applied or high-dose biochar
Microplastic Mitigation	Adsorbs microplastic particles and toxic additives, limiting their mobility and bioavailability	Limited evidence on long-term MP–biochar interactions or degradation pathways
Environmental Impact	Sequesters carbon; reduces greenhouse gas emissions from soil	Incomplete pyrolysis may release harmful VOCs or PAHs
Economic Feasibility	Utilizes agricultural waste; potential for circular economy	High initial production and application costs; limited access to pyrolysis technology in developing regions

### 9.3 Need for long-term field studies and standardized methodologies

To fully understand the impact of microplastics on nitrogen cycling and wheat productivity, long-term field studies are essential. These studies should track microplastic accumulation in agricultural soils over multiple growing seasons and evaluate its effects on soil structure, microbial communities, and nutrient availability. Standardized methodologies for microplastics detection and quantification must also be developed to improve data reliability and facilitate cross-study comparisons (**Table 11**). Field-based research should also explore the interactions between microplastics and different nitrogen fertilizers under varying climate conditions, soil types, and agricultural practices. Another crucial aspect is investigating mitigation strategies, such as biochar application, to determine their effectiveness in reducing microplastics-related disruptions in soil nutrient cycling. Additionally, future research should examine the potential transfer of microplastics from soil to crops and assess their implications for human health and food safety. By addressing these knowledge gaps, researchers can provide clearer guidance on sustainable agricultural practices that minimize microplastic contamination and ensure long-term soil fertility.

**Table 11:** Comparative assessment of proposed microplastic mitigation strategies in agroecosystems.

Strategy	Effectiveness	Limitations	Potential risks	Scalability/Cost
Biochar Application	- High MP sorption - Improves N retention - Buffers pH & microbial health	- Feedstock dependent - Long-term effects uncertain	- Heavy metal/PAH contamination - Microbial shifts - SOM priming	Medium–High cost; scalable with quality assurance
Compost/FYM	- Enhances microbial resilience - Boosts nutrient cycling	- May contain MPs - Variable nutrient content	- Reintroduction of MPs - GHG emissions if unmanaged	Low cost; highly scalable but needs input quality control
Slow-Release/Coated Fertilizers	- Reduces NH_3_ loss - Synchronizes N release with crop demand	- Costly - Limited MP-specific trials	- Accumulation of coating residues - Potential over-application	High cost; moderate scalability
Microbial Inoculants/Bioremediation	- Potential MP degradation - Restores soil microbiome	- Environment-specific performance - Competition with native microbes	- Ecosystem disruption - Horizontal gene transfer from engineered strains	Variable cost; low–moderate scalability
Cover Cropping & Conservation Tillage	- Indirect MP mitigation - Improves SOC & microbial diversity	- Slower effect - Less effective on legacy MP contamination	- Minimal risk if well managed	Low cost; high scalability
Biochar–Compost–Urea Blends	- Synergistic effects on N retention, MP binding, and microbial recovery	- Requires site-specific calibration - Needs compatibility of components	- Interaction effects unpredictable without field data	Moderate cost; promising but field validation required

## 10 Future perspectives

As concerns about microplastic pollution in agriculture continue to grow, it is imperative to develop strategies that minimize its impact on soil health and crop productivity. Future research should focus on identifying sustainable agricultural practices that reduce plastic use while maintaining soil fertility and nitrogen availability. This includes promoting the use of biodegradable mulch films, improving waste management systems to limit plastic pollution, and adopting precision farming techniques that optimize nitrogen fertilizer application. Enhancing soil organic matter through practices such as cover cropping and composting may also help mitigate the negative effects of microplastics on soil microbial activity and nutrient cycling. Additionally, integrating biochar into farming systems has shown promise in reducing ammonia volatilization and improving nitrogen use efficiency, making it a potential solution for addressing microplastics-related soil disruptions.

### 10.1 Recommendations for sustainable agricultural practices

To minimize microplastic contamination in agricultural soils, farmers should consider reducing the reliance on plastic-based products, such as synthetic mulch films, and explore eco-friendly alternatives. Improved waste management practices, such as proper disposal and recycling of agricultural plastics, are essential to prevent microplastic accumulation in farmlands. The adoption of organic amendments, including farmyard manure and compost, can enhance soil microbial activity and nutrient retention, counteracting some of the negative effects of microplastics. Additionally, biochar application has been identified as a promising approach to improve nitrogen retention in soil and mitigate ammonia volatilization (**Table 12**). Precision nitrogen management, including the use of slow-release and biochar-coated fertilizers, can further enhance nutrient efficiency while reducing the risk of nitrogen loss due to microplastic interference. Policymakers should also encourage research on biodegradable agricultural plastics and support the development of innovative solutions that balance productivity with environmental sustainability.

**Table 12:** Cost–benefit framework for biochar application in agricultural systems affected by microplastics.

Dimension	Potential benefits	Potential costs/constraints
Environmental	- Enhanced soil carbon sequestration - Reduced MP toxicity and mobility - Lower GHG emissions	- Possible introduction of PAHs or VOCs (if pyrolysis is suboptimal)
Agronomic	- Improved crop productivity through better nutrient and water retention - Stabilization of soil microbial functions	- Yield benefits vary with biochar type, dose, and crop species
Economic	- Use of local organic waste - Long-term fertility improvements	- Pyrolysis equipment and labor costs - Transport and field application expenses
Social/Institutional	- Promotes sustainable waste recycling - Encourages farmer innovation	- Lack of policy support or incentives - Knowledge and training gaps
Research/Regulatory	- Potential for integration into climate-smart agriculture policies	- Inadequate long-term field data on MP-biochar interactions

### 10.2 Policy implications and strategies for reducing microplastic contamination

Regulatory frameworks should be established to monitor and control microplastic contamination in agricultural soils. Governments and environmental agencies should implement policies that promote the responsible use of plastic materials in farming, including restrictions on non-biodegradable plastic mulch films and incentives for adopting sustainable alternatives. Additionally, agricultural extension programs should educate farmers on the risks associated with microplastic pollution and provide training on best management practices for reducing plastic waste. Investment in research and development of biodegradable plastics and eco-friendly soil amendments should be prioritized to create viable alternatives that do not compromise soil health. Strengthening collaboration between scientists, policymakers, and farmers is crucial to developing practical and effective solutions to address microplastic pollution in agriculture.

## 11 Conclusion

This study highlights the significant impact of microplastic pollution on the nitrogen cycle and ammonia volatilization in agricultural soils, with direct consequences for soil health and crop productivity. Microplastics, particularly polyethylene (PE), polyvinyl chloride (PVC), and polypropylene (PP), influence nitrogen transformations, disrupt microbial communities, and alter soil physical and chemical properties. The findings indicate that microplastics reduce nitrogen use efficiency (NUE) by interfering with processes such as nitrification, denitrification, and nitrogen mineralization, leading to increased ammonia volatilization and nitrogen losses. The extent of these effects depends on microplastics’ size, shape, concentration, and soil type, reinforcing the complexity of their interactions within agricultural ecosystems.

The study also examined the interaction of microplastics with various nitrogen sources, including urea, farmyard manure (FYM), biochar-coated urea, and polymer-coated urea. Results suggest that microplastics adsorb nitrogen compounds, reduce fertilizer efficiency, and alter soil microbial activity, ultimately affecting plant nutrient uptake. While organic amendments such as FYM and biochar offer potential remediation strategies, microplastic contamination within these fertilizers may contribute to long-term soil degradation. The effectiveness of biochar-based solutions was also evaluated, showing that biochar can mitigate microplastics-induced disruptions by improving soil structure, enhancing microbial diversity, and reducing ammonia volatilization. However, further field-based research is needed to optimize biochar applications and assess their long-term impact on nitrogen cycling and crop yield.

Given the increasing presence of microplastics in agricultural soils, urgent measures are required to mitigate their effects and develop sustainable management strategies. Future research should focus on long-term field studies, assessing the cumulative impact of microplastics on soil health, nitrogen transformations, and food safety. Additionally, policies should be implemented to regulate plastic use in agriculture, promote biodegradable alternatives, and encourage improved waste management practices. Addressing these challenges through an integrated approach, combining scientific research, sustainable agricultural practices, and policy interventions, is essential to safeguarding soil fertility, crop productivity, and environmental sustainability in the face of growing microplastic contamination.

### 11.1 Summary of findings

The presence of microplastics in agricultural soils can significantly alter soil properties, nutrient availability, and microbial activity, leading to potential reductions in wheat growth and productivity. Studies indicate that polyethylene (PE), polyvinyl chloride (PVC), and polypropylene (PP) interact with nitrogen fertilizers, influencing nitrogen use efficiency and ammonia volatilization rates. Biochar has been identified as a potential remediation strategy, improving soil structure and mitigating some of the negative effects of microplastic contamination. However, inconsistencies in research methodologies and the lack of long-term field studies highlight the need for further investigation.

### 11.2 Emphasis on the need for integrated approaches to address microplastic pollution

To effectively manage microplastic contamination in agricultural soils, an integrated approach combining scientific research, sustainable agricultural practices, and policy regulations is required. Long-term field studies are needed to assess the cumulative effects of microplastics on soil health and crop productivity. Farmers should adopt environmentally friendly practices, such as reducing plastic use, improving waste management, and incorporating biochar-based amendments to enhance nitrogen retention. Policymakers must implement regulations to limit plastic pollution and support the development of biodegradable alternatives. By addressing these challenges collectively, it is possible to ensure sustainable agricultural production while mitigating the risks posed by microplastic pollution.

## References

[B1] Abalos D. Jeffery S. Sanz-Cobena A. Guardia G. Vallejo A. (2014). Meta-analysis of the effect of urease and nitrification inhibitors on crop productivity and nitrogen use efficiency. Agriculture Ecosyst. Environ. 189, 136–144. doi: 10.1016/j.agee.2014.03.036

[B2] Ali N. Khan M. H. Ali M. Sidra S. Ahmad S. Khan A.. (2023). Insight into microplastics in the aquatic ecosystem: Properties, sources, threats, and mitigation strategies. Sci. Total Environ. 913, 169489. doi: 10.1016/j.scitotenv.2023.169489, PMID: 38159747

[B3] Arias-Andres M. Kettner M. T. Miki T. Grossart H.-P. (2018). Microplastics: New substrates for heterotrophic activity contribute to altering organic matter cycles in aquatic ecosystems. Sci. Total Environ. 635, 1152–1159. doi: 10.1016/j.scitotenv.2018.04.199, PMID: 29710570

[B4] Avinash G. P. Karthick Raja Namasivayam S. Arvind Bharani R. S. (2023). A critical review on occurrence, distribution, environmental impacts and biodegradation of microplastics. J. Environ. Biol. 44, 655–664. doi: 10.22438/jeb/44/5/mrn-5099

[B5] Avnimelech Y. Laher M. (1977). Ammonia volatilization from soils: equilibrium considerations. Soil Sci. Soc. America J. 41, 1080–1084. doi: 10.2136/sssaj1977.03615995004100060013x

[B6] Azeem I. Adeel M. Shakoor N. Zain M. Bibi H. Azeem K.. (2024). Co-exposure to tire wear particles and nickel inhibits mung bean yield by reducing nutrient uptake. Environ. Sci.: Proc. Impacts 26, 832–842., PMID: 38619070 10.1039/d4em00070f

[B7] Bai R. Liu H. Cui J. Wu Y. Guo X. Liu Q.. (2024). The characteristics and influencing factors of farmland soil microplastic in Hetao Irrigation District, China. J. Hazardous Materials 465, 133472. doi: 10.1016/j.jhazmat.2024.133472, PMID: 38219587

[B8] Boots B. Russell C. W. Green D. S. (2019). Effects of microplastics in soil ecosystems: above and below ground. Environ. Sci. Technol. 53, 11496–11506. doi: 10.1021/acs.est.9b03304, PMID: 31509704

[B9] Büks F. Kaupenjohann M. (2020). Global concentrations of microplastic in soils, a review. Soil Discussions 2020, 1–26.

[B10] Cai X. Chen H. Huang B. (2022). Analysis on advances and characteristics of microplastic pollution in China’s lake ecosystems. Ecotoxicology and Environmental Safety 232, 113254., PMID: 35104781 10.1016/j.ecoenv.2022.113254

[B11] Chantigny M. H. Côté D. Massé D. Rochette P. Angers D. A. (2004). Ammonia volatilization and selected soil characteristics following application of anaerobically digested pig slurry. Soil Sci. Soc. America J. 68, 306–312. doi: 10.2136/sssaj2004.3060

[B12] Chen H. Wang Y. Ma Y. Zhang Y. (2022). Biochar-microplastic interactions in soil: Effects on nutrient cycling and microbial communities. J. Hazardous Materials 425, 127939. doi: 10.1016/j.jhazmat.2021.127939, PMID: 34893377

[B13] Chen L. Wang S. Wang P. Xue H. Mei S. Hua Z.. (2021). Comparison of nitrogen loss weight in ammonia volatilization, runoff, and leaching between common and slow-release fertilizer in paddy field. Water Air Soil pollut. 232, 118733. doi: 10.1007/s11270-021-05083-6

[B14] Chen X. Xie Y. Wang J. Shi Z. Zhang J. Wei H.. (2023). Presence of different microplastics promotes greenhouse gas emissions and alters the microbial community composition of farmland soil. Sci. Total Environ. 879, 162967. doi: 10.1016/j.scitotenv.2023.162967, PMID: 36948309

[B15] Cocozza P. Serranti S. Setini A. Cucuzza P. Bonifazi G. (2024). Monitoring of contamination by microplastics on sandy beaches at Vulcano Island (Sicily, Italy) by hyperspectral imaging. Environ. Sci. pollut. Res. Int. doi: 10.1007/s11356-024-34972-6, PMID: 39320598 PMC12325522

[B16] Collignon A. Hecq J.-H. Galgani F. Collard F. Goffart A. (2013). Annual variation in neustonic micro- and meso-plastic particles and zooplankton in the Bay of Calvi (Mediterranean–Corsica). Mar. pollut. Bull. 79, 293–298. doi: 10.1016/j.marpolbul.2013.11.023, PMID: 24360334

[B17] Colzi I. Marangoni R. Cecchini M. (2021). The effect of microplastics on the growth and health of pumpkins. Environ. pollut. 269, 115804. doi: 10.1016/j.envpol.2020.115804, PMID: 33065362

[B18] Corradini F. Casado F. Leiva V. Huerta-Lwanga E. Geissen V. (2020). Microplastics occurrence and frequency in soils under different land uses on a regional scale. Sci. Total Environ. 752, 141917. doi: 10.1016/j.scitotenv.2020.141917, PMID: 32892050

[B19] Corradini F. Meza P. Eguiluz R. Casado F. Huerta-Lwanga E. Geissen V. (2019). Evidence of microplastic accumulation in agricultural soils from sewage sludge disposal. Sci. Total Environ. 671, 411–420., PMID: 30933797 10.1016/j.scitotenv.2019.03.368

[B20] D’Avignon G. Gregory-Eaves I. Ricciardi A. (2021). Microplastics in lakes and rivers: an issue of emerging significance to limnology. Environ. Rev. 30, 228–244. doi: 10.1139/er-2021-0048

[B21] De Sá L. C. S. (2018). A critical review of the impact of microplastics in the environment. Environ. Sci. Technol. 52, 10628–10635. doi: 10.1021/acs.est.8b03010, PMID: 30092628

[B22] de Souza MaChado A. A. Lau C. W. Till J. Kloas W. Lehmann A. Becker R.. (2018). Impacts of microplastics on the soil biophysical environment. Environ. Sci. Technol. 52, 9656–9665. doi: 10.1021/acs.est.8b02212, PMID: 30053368 PMC6128618

[B23] de Souza MaChado A. A. Lau C. W. Till J. Kloas W. Lehmann A. Becker R.. (2019). Impacts of microplastics on the soil biophysical environment. Environ. Sci. Technol. 53, 6044–6052. doi: 10.1021/acs.est.9b01339, PMID: 30053368 PMC6128618

[B24] Dindar E. (2024). The effect of N mineralization, nitrification and ammonification rates in soils contaminated with microplastics. Water Air Soil pollut. 235, 699.

[B25] Dong Y. Lau P. W. Dong B. Zou Z. Yang Y. Wen B.. (2019). Trends in physical fitness, growth, and nutritional status of Chinese children and adolescents: a retrospective analysis of 1· 5 million students from six successive national surveys between 1985 and 2014. The Lancet Child & Adolescent Health 3, 871–880., PMID: 31582349 10.1016/S2352-4642(19)30302-5

[B26] Duan X. Chen X. Shi L. Cao Y. Liang Y. Huang C.. (2025). Functionality-dependent removal efficiency and mechanisms of polystyrene microplastics by a robust magnetic biochar. J. Environ. Chem. Eng. 13, 115509. doi: 10.1016/j.jece.2025.115509

[B27] Elbasiouny H. Albeialy N. O. Aeash N. R. Sharaf-Eldin A. M. Elbanna B. A. Zedan A.. (2023). Potential effect of biochar on soil properties, microbial activity and *vicia faba* properties affected by microplastics contamination. Agronomy 13, 149. doi: 10.3390/agronomy13010149

[B28] Galloway T. S. Cole M. Lewis C. (2017). How microplastics interact with other pollutants. Environ. Toxicol. Chem. 36, 1224–1233. doi: 10.1002/etc.3796, PMID: 28300282

[B29] Gao F. Li J. Sun C. Zhang L. Jiang F. Cao W.. (2019). Study on the capability and characteristics of heavy metals enriched on microplastics in marine environment. Mar. pollut. Bull. 144, 61–67. doi: 10.1016/j.marpolbul.2019.04.039, PMID: 31180007

[B30] Guo X. Chen C. Wang J. (2019). Sorption of sulfamethoxazole onto six types of microplastics. Chemosphere 228, 300–308. doi: 10.1016/j.chemosphere.2019.04.155, PMID: 31035168

[B31] Guo Z. Chen W. Wang Z. Yang X. Geissen V. Li G.. (2022). Soil texture is an important factor determining how microplastics affect soil hydraulic characteristics. Environ. Int. 165, 107293. doi: 10.1016/j.envint.2022.107293, PMID: 35609499

[B32] Guo J. Fan T. Chen X. Wang Y. Cui Z. (2016). Designing corn management strategies for high yield and high nitrogen use efficiency. Agron. J. 108, 922–929. doi: 10.2134/agronj2015.0435

[B33] Habib S. Iruthayam A. Alias S. A. Smykla J. Abd Shukor M. Y. Yasid N. A. (2020). Biodeterioration of untreated polypropylene microplastic particles by antarctic bacteria. Polymers 12, 2616. doi: 10.3390/polym12112616, PMID: 33172014 PMC7694613

[B34] Hao J. Feng Y. Wang X. Yu Q. Zhang F. Yang G.. (2022). Soil microbial nitrogen-cycling gene abundances in response to crop diversification: A meta-analysis. Sci. Total Environ. 838, 156621. doi: 10.1016/j.scitotenv.2022.156621, PMID: 35691356

[B35] Hasan M. M. Tarannum M. N. (2025). Adverse impacts of microplastics on soil physicochemical properties and crop health in agricultural systems. J. Hazardous Materials Adv. 17, 100528. doi: 10.1016/j.hazadv.2025.100528

[B36] Heiss E. M. Fulweiler R. W. (2016). Coastal water column ammonium and nitrite oxidation are decoupled in summer. Estuarine Coast. Shelf Sci. 178, 110–119. doi: 10.1016/j.ecss.2016.06.002

[B37] Huang W. Huang G. An S. Zhang H. (2022). Microplastic-induced changes in microbial nitrogen transformation and organic matter decomposition. J. Hazardous Materials 435, 128986. doi: 10.1016/j.jhazmat.2022.128986, PMID: 35487002

[B38] Huang J. B. Zeng G. S. Li X. S. Tong H. Cheng X. C. (2018). “Theoretical studies on bond dissociation enthalpies for model compounds of typical plastic polymers,” in IOP Conference Series: Earth and Environmental Science, Vol. 167. 012029. doi: 10.1088/1755-1315/167/1/012029

[B39] Huang P. Zhang Y. Hussain N. Lan T. Chen G. Tang X.. (2023). A bibliometric analysis of global research hotspots and progress on microplastics in soil–plant systems. Environ. pollut. 341, 122890. doi: 10.1016/j.envpol.2023.122890, PMID: 37944892

[B40] Huggins D. R. Pan W. L. (2003). Key indicators for assessing nitrogen use efficiency in cereal-based agroecosystems. J. Crop Production 8, 157–185. doi: 10.1300/j144v08n01_07

[B41] Ingraffia R. Bagarello V. Iovino M. Lehmann A. Carollo F. G. Rillig M. C.. (2022). Polyester microplastic fibers affect soil physical properties and erosion as a function of soil type. SOIL 8, 421–435. doi: 10.5194/soil-8-421-2022

[B42] Jat R. A. Wani S. P. Sahrawat K. L. Singh P. Dhaka S. R. Dhaka B. L. (2012). Recent approaches in nitrogen management for sustainable agricultural production and eco-safety. Arch. Agron. Soil Sci. 58, 1033–1060. doi: 10.1080/03650340.2011.557368

[B43] Jia W. Karapetrova A. Zhang M. Xu L. Li K. Huang M.. (2022). Automated identification and quantification of invisible microplastics in agricultural soils. Sci. Total Environ. 844, 156853. doi: 10.1016/j.scitotenv.2022.156853, PMID: 35752236

[B44] Jin H. Lin G. Ma M. Wang L. Liu L. (2024). The effects of polystyrene microplastics and copper ion co-contamination on the growth of rice seedlings. Nanomaterials. doi: 10.3390/nano15010017, PMID: 39791777 PMC11723024

[B45] Komatsuzaki M. Ohta H. (2007). Soil management practices for sustainable agro-ecosystems. Sustainability Sci. 2, 103–120. doi: 10.1007/s11625-006-0014-5

[B46] Kothari M. Nimje P. Mistry D. Jagtap K. (2024). Microplastics pollution control in agricultural soils. Microplastics pollut.

[B47] Kumar A. Mishra S. Pandey R. Yu Z. G. Kumar M. Khoo K. S.. (2022). Microplastics in terrestrial ecosystems: Un-ignorable impacts on soil characterises, nutrient storage and its cycling. TrAC Trends Analytical Chem. 158, 116869. doi: 10.1016/j.trac.2022.116869

[B48] Lakshmikanthan D. Punithavathi V. R. (2024). Combined effects of micro-/nanoplastics and humic substances on *Allium sativum* and importance of humic substances in alleviating toxicity. Sciforum.

[B49] Lee Y.-J. Im E.-C. Lee G. Hong S.-C. Lee C.-G. Park S.-J. (2024). Comparison of ammonia volatilization in paddy and field soils fertilized with urea and ammonium sulfate during rice, potato, and Chinese cabbage cultivation. Atmospheric pollut. Res. 15, 102049. doi: 10.1016/j.apr.2024.102049

[B50] Lei L. Wu S. Lu S. Liu M. Song Y. Fu Z.. (2017). Microplastic particles cause intestinal damage and other adverse effects in zebrafish *Danio rerio* and nematode *Caenorhabditis elegans* . Sci. Total Environ. 619–620, 1–8. doi: 10.1016/j.scitotenv.2017.11.103, PMID: 29136530

[B51] Li H. Helmberger M. Tiemann L. K. Billings S. A. (2020a). Microplastic addition alters organic carbon decomposition and microbial community structure. Soil Biol. Biochem. 142, 107701. doi: 10.1016/j.soilbio.2020.107701

[B52] Li J. Yu S. Yu Y. (2022). Effects of microplastics on higher plants: a review. Bulletin of Environmental Contamination and Toxicology 109, 241–265. 35752996 10.1007/s00128-022-03566-8

[B53] Li L. Song K. Yeerken S. Geng S. Liu D. Dai Z.. (2019). Effect evaluation of microplastics on activated sludge nitrification and denitrification. Sci. Total Environ. 707, 135953. doi: 10.1016/j.scitotenv.2019.135953, PMID: 31865070

[B54] Li H. Wang L. Peng Y. Zhang S. Lv S. Li J.. (2020b). Film mulching, residue retention and N fertilization affect ammonia volatilization through soil labile N and C pools. Agriculture Ecosyst. Environ. 308, 107272. doi: 10.1016/j.agee.2020.107272

[B55] Lian J. Wu J. Zeb A. Zheng S. Ma T. Peng F.. (2020). Do polystyrene nanoplastics affect the toxicity of cadmium to wheat (Triticum aestivum L.)? Environ. Poll. 263, 114498., PMID: 33618453 10.1016/j.envpol.2020.114498

[B56] Lithner D. Larsson Å. Dave G. (2011). Environmental and health hazard ranking and assessment of plastic polymers based on chemical composition. Science of the total environment 409, 3309–3324., PMID: 21663944 10.1016/j.scitotenv.2011.04.038

[B57] Liu Q. Chen X. Wu K. Fu X. (2015). Nitrogen signaling and use efficiency in plants: what’s new? Curr. Opin. Plant Biol. 27, 192–198. doi: 10.1016/j.pbi.2015.08.002, PMID: 26340108

[B58] Liu E. K. He W. Yan C. R. (2022b). Microplastic pollution and its effects on soil environment: A review. Sci. Total Environ. 822, 153599. doi: 10.1016/j.scitotenv.2022.153599, PMID: 35114243

[B59] Liu Y. Hu B. Chu C. (2022c). Toward improving nitrogen use efficiency in rice: Utilization, coordination, and availability. Curr. Opin. Plant Biol. 71, 102327. doi: 10.1016/j.pbi.2022.102327, PMID: 36525788

[B60] Liu X. Xu J. Zhao S. Chen J. (2023). Impacts of polymer-coated fertilizers and microplastics on soil structure and nitrogen transformation. Agric. Syst. 203, 103513. doi: 10.1016/j.agsy.2022.103513

[B61] Liu D. Zheng Y. Chen L. Wen D. (2022a). Prevalence of small-sized microplastics in coastal sediments detected by multipoint confocal micro-Raman spectrum scanning. Sci. Total Environ. 831, 154741. doi: 10.1016/j.scitotenv.2022.154741, PMID: 35339562

[B62] Liu Y. Liu J. Xia H. Zhang X. Fontes-Garfias C. R. Swanson K. A.. (2021). Neutralizing activity of BNT162b2-elicited serum. N. Engl. J. Med. 384, 1466–1468., PMID: 33684280 10.1056/NEJMc2102017 PMC7944950

[B63] Lu P. Yan Z. Lu G. (2021). Influence of microplastics on nitrogen cycle in different environments. Res. Environ. Sci. 34, 2563–2570.

[B64] Mandal S. Thangarajan R. Bolan N. S. Sarkar B. Khan N. Ok Y. S.. (2015). Biochar-induced concomitant decrease in ammonia volatilization and increase in nitrogen use efficiency by wheat. Chemosphere 142, 120–127. doi: 10.1016/j.chemosphere.2015.04.086, PMID: 25959224

[B65] Marcharla E. Vinayagam S. Gnanasekaran L. Soto-Moscoso M. Chen W. H. Thanigaivel S.. (2024). Microplastics in marine ecosystems: A comprehensive review of biological and ecological implications and its mitigation approach using nanotechnology for the sustainable environment. Environ. Res. 256, 119181. doi: 10.1016/j.envres.2024.119181, PMID: 38768884

[B66] Martínez-Espinosa R. M. Watmough N. J. Richardson D. J. Cole J. A. (2011). Enzymology and ecology of the nitrogen cycle. Biochem. Soc. Trans. 39, 175–178. doi: 10.1042/bst0390175, PMID: 21265768

[B67] Mbedzi R. Murungweni F. M. Wasserman R. J. Dalu T. Cuthbert R. N. (2020). Spatiotemporal variation in microplastic contamination along a subtropical reservoir shoreline. Environ. Sci. pollut. Res. 27, 23880–23887. doi: 10.1007/s11356-020-08640-4, PMID: 32301080

[B68] Meng F. Fan T. Yang X. Riksen M. Xu M. Geissen V. (2020). Effects of plastic mulching on the accumulation and distribution of macro and micro plastics in soils of two farming systems in Northwest China. PeerJ 8, e10375. doi: 10.7717/peerj.10375, PMID: 33344073 PMC7718786

[B69] Meng F. Yang X. Riksen M. Xu M. Geissen V.. (2021). Response of common bean (Phaseolus vulgaris L.) growth to soil contaminated with microplastics. Sci. Total Environ. 755, 142516., PMID: 33045612 10.1016/j.scitotenv.2020.142516

[B70] Miao J. Huang W. Pan R. (2023). Research progress and hotspot analysis of soil microplastics: a bibliometrics-based review. Frontiers in Environmental Science 11, 1297646.

[B71] Monib A. W. Fahmawi S. M. S. Barai S. M. Baseer A. Q. Alikhail M. Niazi P.. (2024). Nitrogen cycling dynamics: Investigating volatilization and its interplay with N_2_ fixation. J. Res. Appl. Sci. Biotechnol. 3, 17–31. doi: 10.55544/jrasb.3.1.4

[B72] Moreau D. Philippot L. Jones D. L. Bardgett R. D. Finlay R. D. (2019). A plant perspective on nitrogen cycling in the rhizosphere. Funct. Ecol. 33, 540–552. doi: 10.1111/1365-2435.13303

[B73] Mota L. S. O. de Oliveira P. C. O. Peixoto B. S. Bezerra E. S. de Moraes M. C. (2025). Biochar applications in microplastic and nanoplastic removal: mechanisms and integrated approaches. Environ. Science: Water Res. Technol. 11, 222–241. doi: 10.1039/D3EW00666D

[B74] Nihei Y. Ota H. Tanaka M. Kataoka T. Kashiwada J. (2023). Comparison of concentration, shape, and polymer composition between microplastics and mesoplastics in Japanese river waters. Water Res. 249, 120979. doi: 10.1016/j.watres.2023.120979, PMID: 38086208

[B75] Okoffo E. D. Mueller J. F. Tscharke B. J. Gallen M. Ribeiro F. Thomas K. V.. (2020). Identification and quantification of selected plastics in biosolids by pressurized liquid extraction combined with double-shot pyrolysis gas chromatography-mass spectrometry. Sci. Total Environ. 715, 136924. doi: 10.1016/j.scitotenv.2020.136924, PMID: 32007891

[B76] Piehl S. Leibner A. Löder M. G. J. Dris R. Bogner C. Laforsch C. (2018). Identification and quantification of macro- and microplastics on agricultural farmland. Sci. Rep. 8, 17950. doi: 10.1038/s41598-018-36172-y, PMID: 30560873 PMC6299006

[B77] Praveena S. M. Nafisyah A. L. Hisham M. A. F. I. (2023). Microplastics pollution in agricultural farms soils: Preliminary findings from tropical environment (Klang Valley, Malaysia). Environ. Monit. Assess. 195, 108285. doi: 10.1007/s10661-023-11250-5, PMID: 37160548

[B78] Qi R. Jones D. L. Li Z. Liu Q. Yan C. (2019a). Behavior of microplastics and plastic film residues in the soil environment: A critical review. Sci. Total Environ. 703, 134722. doi: 10.1016/j.scitotenv.2019.134722, PMID: 31767311

[B79] Qi Y. Yang X. Pelaez A. M. Huerta Lwanga E. (2019b). Macro- and microplastics in soil–plant systems: Effects of plastic mulch film residues on wheat (*Triticum aestivum*) growth. Sci. Total Environ. 645, 1048–1056., PMID: 30248830 10.1016/j.scitotenv.2018.07.229

[B80] Qi Y. Yang X. Pelaez A. M. Huerta Lwanga E. (2020). Microplastic pollution and its effect on wheat growth and nitrogen uptake. Sci. Total Environ. 707, 135774.

[B81] Qiu Y. Zhou S. Zhang C. Zhou Y. Qin W. (2022). Soil microplastic characteristics and the effects on soil properties and biota: A systematic review and meta-analysis. Environ. pollut. 313, 120183. doi: 10.1016/j.envpol.2022.120183, PMID: 36126769

[B82] Rahut D. B. Shimly S. Rajendrakumar S. (2025). Far-reaching impact of microplastics on agricultural systems: Options for mitigation and adaptation. Land Degradation Dev.

[B83] Rao D. L. N. Batra L. (1983). Ammonia volatilization from applied nitrogen in alkali soils. Plant Soil 70, 219–228. doi: 10.1007/bf02374782

[B84] Ren X. Sun H. Tang J. Wang L. (2021). Combined effects of microplastics and biochar on the removal of polycyclic aromatic hydrocarbons and phthalate esters and its potential microbial ecological mechanism. Front. Microbiol. 12. doi: 10.3389/fmicb.2021.647766, PMID: 33995304 PMC8120302

[B85] Rillig M. C. (2012). Microplastics in soil and their impact. Environ. Sci. Technol. 46, 6453–6454. doi: 10.1021/es302011r, PMID: 22676039

[B86] Rillig M. C. Lehmann A. de Souza Machado A. A. Yang G. (2019). Microplastic effects on plants. New phytologist 223, 1066–1070. 30883812 10.1111/nph.15794

[B87] Rillig M. C. Lehmann J. de Souza MaChado A. A. Yang G. (2021). Microplastic effects on carbon cycling processes in soils. Sci. Adv. 7, eabe2515. doi: 10.1371/journal.pbio.3001130, PMID: 33784293 PMC8009438

[B88] Riveros G. Urrutia H. Araya J. Zagal E. Schoebitz M. (2022). Microplastic pollution on the soil and its consequences on the nitrogen cycle: A review. Environ. Sci. pollut. Res. 29, 7997–8011. doi: 10.1007/s11356-021-17681-2, PMID: 34825330

[B89] Saqib S. Ullah F. Wang P. Y. Zhao L. Ashraf M. Khan A. (2025). Microplastics unveiled: Understanding their toxicological impact on terrestrial ecosystems. iScience. 10.1016/j.isci.2025.111879 PMC11848805 39995877

[B90] Schlesinger W. H. Peterjohn W. T. (1991). Processes controlling ammonia volatilization from Chihuahuan desert soils. Soil Biol Biochem 23, 637–642.

[B91] Scott N. Porter A. Santillo D. Simpson H. Lloyd-Williams S. Lewis C. (2019). Particle characteristics of microplastics contaminating the mussel *Mytilus edulis* and their surrounding environments. Mar. pollut. Bull. 146, 125–133. doi: 10.1016/j.marpolbul.2019.05.041, PMID: 31426140

[B92] Sha Z. Ma X. Liu H. Wang J. Lv T. Goulding K.. (2023). Crop-specific ammonia volatilization rates and key influencing factors in the upland of China – A data synthesis. J. Environ. Manage. 336, 117676. doi: 10.1016/j.jenvman.2023.117676, PMID: 36967697

[B93] Shan L. He Y. Chen J. Huang Q. Wang H. (2015). Ammonia volatilization from a Chinese cabbage field under different nitrogen treatments in the Taihu Lake Basin, China. J. Environ. Sci. 38, 14–23. doi: 10.1016/j.jes.2015.04.028, PMID: 26702964

[B94] Stabnikova O. Stabnikov V. Klavins L. Marinin A. Vaseashta A. Klavins M. (2021). Microbial life on the surface of microplastics in natural waters. Appl. Sci. 11, 11692. doi: 10.3390/app112411692

[B95] Su X. Qian F. Bao Y. (2024). The effect of bulk-biochar and nano-biochar amendment on the removal of antibiotic resistance genes in microplastic contaminated soil. Environ. Res. 240, 117488. doi: 10.1016/j.envres.2023.117488, PMID: 37907163

[B96] Sun Y. Ji J. Tao J. Yang Y. Wu D. Han L.. (2022). Current advances in interactions between microplastics and dissolved organic matters in aquatic and terrestrial ecosystems. TrAC Trends Analytical Chem. 158, 116882. doi: 10.1016/j.trac.2022.116882

[B97] Sun L. Liu Y. Feng Y. Fan Z. Jiang L. Lu C. (2025). Aged polylactic acid microplastics with ultraviolet irradiation stunted pakchoi germination and growth with cadmium in hydroponics. J. Hazardous Materials. doi: 10.1016/j.ecoenv.2025.117696, PMID: 39788031

[B98] Sun Y. Ren X. Pan J. Zhang Z. Tsui T. H. Luo L.. (2020). Effect of microplastics on greenhouse gas and ammonia emissions during aerobic composting. Sci. Total Environ. 737, 139856. doi: 10.1016/j.scitotenv.2020.139856, PMID: 32563113

[B99] Surendran P. Stewart I. D. Au Yeung V. P. Pietzner M. Raffler J. Wörheide M. A.. (2022). Rare and common genetic determinants of metabolic individuality and their effects on human health. Nat. Med. 28, 2321–2332. 36357675 10.1038/s41591-022-02046-0 PMC9671801

[B100] Surendran U. Jayakumar M. Raja P. Gopinath G. Chellam P. V. (2023). Microplastics in terrestrial ecosystem: Sources and migration in soil environment. Chemosphere 318, 137946. doi: 10.1016/j.chemosphere.2023.137946, PMID: 36708782

[B101] Tan X. Liu Y. Zeng G. Wang X. Hu X. Gu Y.. (2015). Application of biochar for the removal of pollutants from aqueous solutions. Chemosphere 125, 70–85. doi: 10.1016/j.chemosphere.2014.12.058, PMID: 25618190

[B102] Tang S. Lin L. Wang X. (2020). Pb (II) uptake onto nylon microplastics: interaction mechanism and adsorption performance. Journal of hazardous materials 386, 121960., PMID: 31893555 10.1016/j.jhazmat.2019.121960

[B103] Tariq M. Khan I. Jho E. H. Salam A. Iqbal B. Li G.. (2024). Microplastic contamination in the agricultural soil: Mitigation strategies, heavy metals contamination, and impact on human health: A review. Plant Cell Rep. 43, 65. doi: 10.1007/s00299-024-03162-6, PMID: 38341396

[B104] Thompson R. B. Meisinger J. J. (2002). Management factors affecting ammonia volatilization from land-applied cattle slurry in the Mid-Atlantic USA. J. Environ. Qual. 31, 1329–1338. doi: 10.2134/jeq2002.1329, PMID: 12175054

[B105] Twardowska I. (2020). How microplastics affect soil, plants, and microorganisms. Soil Sci. Soc. America J. 84, 1571–1584. doi: 10.1002/saj2.20048

[B106] Ventura W. B. Yoshida T. (1977). Ammonia volatilization from a flooded tropical soil. Plant Soil 46, 521–531. doi: 10.1007/bf00015911

[B107] Vlek P. L. G. Craswell E. T. (1979). Effect of nitrogen source and management on ammonia volatilization losses from flooded rice-soil systems. Soil Sci. Soc. America J. 43, 352–358. doi: 10.2136/sssaj1979.03615995004300020023x

[B108] Wang J. Coffin S. Sun C. Scholes C. Swift S. (2020). Interactions between microplastics and soil nutrients: Impacts on nitrogen cycling and availability. Environ. pollut. 263, 1144.

[B109] Wang Y. Joseph S. Chen C. Qi X. Mitchell D. R. G. Si H.. (2023). Goethite-enriched biochar mitigates soil emissions of CO_2_ during arsenic passivation: Effect and mechanisms. Chem. Eng. J. 476, 146542. doi: 10.1016/j.cej.2023.146542

[B110] Wang Y. Wang X. Li Y. Liu Y. Sun Y. Xia S.. (2021). Effects of coexistence of tetracycline, copper and microplastics on the fate of antibiotic resistance genes in manured soil. Sci. Total Environ. 790, 148087. doi: 10.1016/j.scitotenv.2021.148087, PMID: 34091329

[B111] Wang H. Wang D. Zheng Q. He Y. Yang Q. (2025). *Tris*(2,4-di-*tert*-butylphenyl) phosphate as the key toxicant in aged polyvinyl chloride microplastics to wheat roots. ACS Agric. Sci. Technol. doi: 10.1021/acsagscitech.4c00520

[B112] Wang T. Zhao S. Zhu L. McWilliams J. C. Galgani L. Amin R. M.. (2022). Accumulation, transformation and transport of microplastics in estuarine fronts. Nature Reviews Earth & Environment 3, 795–805.

[B113] Wright S. L. Thompson R. C. Galloway T. S. (2013). How microplastics physically affect marine life. Environ. pollut. 178, 483–492. doi: 10.1016/j.envpol.2013.02.031, PMID: 23545014

[B114] Wu Z. Hua Y. Guan C. Luo J. Han Y. Zhang Z. (2019). Low nitrogen enhances nitrogen use efficiency by triggering NO_3_ ^-^ uptake and its long-distance translocation. J. Agric. Food Chem. 67, 6736–6747. doi: 10.1021/acs.jafc.9b02491, PMID: 31184154

[B115] Wu Y. Liu J. Rene E. R. (2017). Periphytic biofilms: A promising nutrient utilization regulator in wetlands. Bioresource Technol. 248, 44–48. doi: 10.1016/j.biortech.2017.07.081, PMID: 28756125

[B116] Wu J. Yang C. Zhao H. Shi J. Liu Z. Li C.. (2023). Efficient removal of microplastics from aqueous solution by a novel magnetic biochar: performance, mechanism, and reusability. Environ. Sci. pollut. Res. 30, 26914–26928. doi: 10.1007/s11356-022-24247-9, PMID: 36374390

[B117] Xie Y. Wang H. Chen Y. Guo Y. Wang C. Cui H.. (2023). Water retention and hydraulic properties of a natural soil subjected to microplastic contaminations and leachate exposures. Science of the Total Environment 901, 166502., PMID: 37619730 10.1016/j.scitotenv.2023.166502

[B118] Xu S. Hu Y. Tahir M. H. Hu W. Zhang P. Tang Y. (2023). Copyrolysis characteristics of polyvinyl chloride, polyethylene and polypropylene based on ReaxFF molecular simulation. Comput. Theor. Chem. 1229, 114350. doi: 10.1016/j.comptc.2023.114350

[B119] Xu B. Liu F. Cryder Z. Huang D. Lu Z. He Y.. (2019). Microplastics in the soil environment: Occurrence, risks, interactions and fate – A review. Crit. Rev. Environ. Sci. Technol. 50, 2175–2222. doi: 10.1080/10643389.2019.1694822

[B120] Xu G. Miller A. J. Fan X. (2012). Plant nitrogen assimilation and use efficiency. Annu. Rev. Plant Biol. 63, 153–182. doi: 10.1146/annurev-arplant-042811-105532, PMID: 22224450

[B121] Xue L. Sun B. Yang Y. Jin B. Zhuang G. Bai Z.. (2021). Efficiency and mechanism of reducing ammonia volatilization in alkaline farmland soil using. Bacillus amyloliquefaciens biofertilizer. Environ. Res. 202, 111672. doi: 10.1016/j.envres.2021.111672, PMID: 34265351

[B122] Yan Y. Gu C. Zhu C. Wang C. Zhu F. Chen Z. (2020). Effect of polyvinyl chloride microplastics on bacterial community and nutrient status in two agricultural soils. Bull. Environ. Contamination Toxicol. 107, 602–609. doi: 10.1007/s00128-020-02900-2, PMID: 32556686

[B123] Yang R. Cheng L. Li Z. Cui Y. Liu J. Xu D.. (2025). Mechanism of microplastics in the reduction of cadmium toxicity in tomato. Ecotoxicology Environ. Saf. doi: 10.1016/j.ecoenv.2024.117621, PMID: 39752910

[B124] Yang Y. Li Z. Yan C. Chadwick D. Jones D. L. Liu E.. (2021). Kinetics of microplastic generation from different types of mulch films in agricultural soil. Sci. Total Environ. 814, 152572. doi: 10.1016/j.scitotenv.2021.152572, PMID: 34954175

[B125] Yang W. H. Silver W. L. Weber K. A. (2012). Nitrogen loss from soil through anaerobic ammonium oxidation coupled to iron reduction. Nat. Geosci. 5, 538–541. doi: 10.1038/ngeo1530

[B126] Yao S. Ni N. Li X. Wang N. Bian Y. Jiang X.. (2023). Interactions between white and black carbon in water: A case study of concurrent aging of microplastics and biochar. Water Res. 238, 120006. doi: 10.1016/j.watres.2023.120006, PMID: 37121197

[B127] Yu J. T. Helm P. A. Diamond M. L. (2023a). Source-specific categorization of microplastics in nearshore surface waters of the Great Lakes. J. Great Lakes Res. 50, 102256. doi: 10.1016/j.jglr.2023.102256

[B128] Yu Y. Varga T. Chowdhury I. Zahid T. M. Battu A. K. Denny A. C.. (2023b). Minimal impacts of microplastics on soil physical properties under environmentally relevant concentrations. Environ. Sci. Technol. 57, 5296–5304. doi: 10.1021/acs.est.2c09822, PMID: 36951544

[B129] Yuan X. Zhang F. Wang Z. (2024). Impacts of micro/nanoplastics combined with graphene oxide on *Lactuca sativa* seeds. Plants. doi: 10.3390/plants13243466, PMID: 39771164 PMC11679930

[B130] Zeng G. Dai M. Liu P. Chen T. Hu L. Luo H.. (2024). Phthalocyanine blue leaching and exposure effects on Microcystis aeruginosa (cyanobacteria) of photoaged microplastics. Journal of Hazardous Materials 469, 133984, PMID: 38460263 10.1016/j.jhazmat.2024.133984

[B131] Zhang Y. Cai C. Gu Y. Shi Y. Gao X. (2022). Microplastics in plant–soil ecosystems: A meta-analysis. Environ. pollut. 308, 119718. doi: 10.1016/j.envpol.2022.119718, PMID: 35809716

[B132] Zhang Z. (2020). A review of how microplastics affect plant growth. Sci. Total Environ. 722, 137817. doi: 10.1016/j.scitotenv.2020.137817, PMID: 32208249

[B133] Zhang Z. Gao S. H. Luo G. Kang Y. Zhang L. Pan Y.. (2021b). The contamination of microplastics in China’s aquatic environment: Occurrence, detection and implications for ecological risk. Environ. pollut. 296, 118737. doi: 10.1016/j.envpol.2021.118737, PMID: 34954308

[B134] Zhang J. Shao Y. Li Z. Han G. Jing X. Wang N.. (2023). Characteristics analysis of plastisphere biofilm and effect of aging products on nitrogen metabolizing flora in microcosm wetlands experiment. J. Hazardous Materials 452, 131336. doi: 10.1016/j.jhazmat.2023.131336, PMID: 37027924

[B135] Zhang G. S. Zhao Y. Sun X. D. Huang Y. (2021a). Microplastics in manure and compost: Occurrence and implications. Sci. Total Environ. 773, 145569. doi: 10.1016/j.scitotenv.2021.145569, PMID: 33592471

[B136] Zhao H. J. Xu J. K. Yan Z. H. Ren H. Q. Zhang Y.. (2020). Microplastics enhance the developmental toxicity of synthetic phenolic antioxidants by disturbing the thyroid function and metabolism in developing zebrafish. Environment International 140, 105750., PMID: 32361124 10.1016/j.envint.2020.105750

[B137] Zheng Y. Cao X. Zhou Y. Li Z. Yang Y. Zhao D.. (2023). Effect of planting salt-tolerant legumes on coastal saline soil nutrient availability and microbial communities. J. Environ. Manage. 345, 118574. doi: 10.1016/j.jenvman.2023.118574, PMID: 37423189

[B138] Zhou J. Gui H. Banfield C. C. Wen Y. Zang H. Dippold M. A.. (2021). The microplastisphere: Biodegradable microplastics addition alters soil microbial community structure and function. Soil Biol. Biochem. 156, 108211. doi: 10.1016/j.soilbio.2021.108211

[B139] Zhou Y. Zhao H. Lu Z. Ren X. Zhang Z. Wang Q. (2023). Synergistic effects of biochar derived from different sources on greenhouse gas emissions and microplastics mitigation during sewage sludge composting. Bioresource Technol. 387, 129556. doi: 10.1016/j.biortech.2023.129556, PMID: 37517712

[B140] Zhou Y. Wang J. Zou M. Yin Q. Qiu Y. Li C.. (2022). Microplastics in urban soils of Nanjing in eastern China: Occurrence, relationships, and sources. Chemosphere 303, 134999., PMID: 35595105 10.1016/j.chemosphere.2022.134999

[B141] Zhuang Q. L. Yuan H. Y. Sun M. Deng H. G. (2025). Biochar-mediated remediation of low-density polyethylene microplastic-polluted soil–plant systems. J. Hazardous Materials. 10.1016/j.jhazmat.2024.137076 39787863

[B142] Zurier H. S. Goddard J. M. (2020). Biodegradation of microplastics in food and agriculture. Curr. Opin. Food Sci. 37, 37–44. doi: 10.1016/j.cofs.2020.09.001

